# Hybrid Polycarbosilane-Siloxane Dendrimers: Synthesis and Properties

**DOI:** 10.3390/polym13040606

**Published:** 2021-02-17

**Authors:** Sergey A. Milenin, Elizaveta V. Selezneva, Pavel A. Tikhonov, Viktor G. Vasil’ev, Alexander I. Buzin, Nikolay K. Balabaev, Andrey O. Kurbatov, Maxim V. Petoukhov, Eleonora V. Shtykova, Lev A. Feigin, Elena A. Tatarinova, Elena Yu. Kramarenko, Sergey N. Chvalun, Aziz M. Muzafarov

**Affiliations:** 1 Enikolopov Institute of Synthetic Polymeric Materials of Russian Academy of Sciences (ISPM RAS), 117393 Moscow, Russia; sellisaveta@gmail.com (E.V.S.); tikhonpa@ispm.ru (P.A.T.); al37919@gmail.com (A.I.B.); tatarinova@ispm.ru (E.A.T.); chvalun@cc.nifhi.ac.ru (S.N.C.); aziz@ispm.ru (A.M.M.); 2 A.N. Nesmeyanov Institute of Organoelement Compounds of Russian Academy of Sciences (INEOS RAS), 119991 Moscow, Russia; viktor@ineos.ac.ru (V.G.V.); kurbatov@polly.phys.msu.ru (A.O.K.); kram@polly.phys.msu.ru (E.Y.K.); 3 Institute of Mathematical Problems of Biology, Keldysh Institute of Applied Mathematics RAS, 142290 Pushchino, Russia; balabaev@impb.psn.ru; 4 Faculty of Physics, Lomonosov Moscow State University, 119991 Moscow, Russia; 5 A.V. Shubnikov Institute of Crystallography of Federal Scientific Research Centre “Crystallography and Photonics” of Russian Academy of Sciences, 119333 Moscow, Russia; maxim@embl-hamburg.de (M.V.P.); eleonora.shtykova@gmail.com (E.V.S.); feigin@ns.crys.ras.ru (L.A.F.); 6 A.N. Frumkin Institute of Physical Chemistry and Electrochemistry of Russian Academy of Sciences, 119071 Moscow, Russia

**Keywords:** dendrimers, synthesis of dendrimers, synthesis of hybrid carbosilane-siloxane structures, thermal properties

## Abstract

A series of carbosilane dendrimers of the 4th, 6th, and 7th generations with a terminal trimethylsilylsiloxane layer was synthesized. Theoretical models of these dendrimers were developed, and equilibrium dendrimer conformations obtained via molecular dynamics simulations were in a good agreement with experimental small-angle X-ray scattering (SAXS) data demonstrating molecule monodispersity and an almost spherical shape. It was confirmed that the glass transition temperature is independent of the dendrimer generation, but is greatly affected by the chemical nature of the dendrimer terminal groups. A sharp increase in the zero-shear viscosity of dendrimer melts was found between the 5th and the 7th dendrimer generations, which was qualitatively identical to that previously reported for polycarbosilane dendrimers with butyl terminal groups. The viscoelastic properties of high-generation dendrimers seem to follow some general trends with an increase in the generation number, which are determined by the regular branching structure of dendrimers.

## 1. Introduction

Dendrimers as a new form of a polymeric substance continue to attract the considerable attention of researchers due to a unique set of chemical and physical properties resulting from their regular tree-like architecture [1,2,3,4,5]. Over the past 30 years, our understanding of the main features of their structure/property relationship has expanded significantly. Intensive experimental and theoretical research has demonstrated that the dualism of the dendrimer nature, expressed in its definition as a macromolecule-particle [6,7], has objective grounds. Accordingly, the term particle is associated not only with the early visualization of the dendrimer shape being close to spherical [8,9], but mainly with the viscosity of their dilute solutions that obeys Einstein’s equation, depending only on the volume fraction of the solute, but not on its molecular weight [5], as well as SAXS data, which are best described by models of monodisperse spherical objects [10,11,12]. The polymer nature of dendrimers can be traced from purely polymer-like dependences of the glass transition temperature on the dendrimer generation number that reaches a plateau after the third or fourth generation [5,7,13,14,15,16], and especially from dendrimer swelling and collapse in dilute solutions, which is controlled by the solvent quality [17,18]. Computer simulations have confirmed the soft molecular nature of dendrimers, demonstrating their internal “life” in dynamics [10,19,20,21,22,23]. 

While the main features of dilute solutions of dendrimers seem to be quite well comprehended, the role of the dendrimer architecture in their intermolecular interactions and resulting properties of their concentrated solutions and melts still remains insufficiently studied. A few researches on dendrimer rheology in bulk were mainly focused on either dendrimers of rather low generations (up to the 5th) [24,25,26] or PAMAM dendrimers containing polar groups [27,28]. In order to assess the effect of the tree-like dendrimer structure itself, it is necessary to choose those systems for study that do not possess any chemical groups interacting via specific interactions, such as, for instance, electrostatic interactions or hydrogen bonding. One example of such systems comprises carbosilane dendrimers that could serve as practically ideal models to monitor the changes in melt properties with increasing dendrimer generation. The first intriguing result was obtained several years ago when extensively studying three homologues series of polybutylcarbosilate dendrimers differing in the functionalities of the core and branching Si atoms (3-3, 4-3 and 4-4 dendrimers). Namely, a transition from a liquid-like to a solid-like behavior of carbosilane dendrimer melts was detected, which was expressed as a second transition on the temperature dependence of the heat capacity [29,30,31,32,33]. This qualitative change of the melt properties was demonstrated for all homologues series and it required some explanation. An important step in quantifying this effect was done in Ref. [34], where rheological properties of the 4-3 carbosilane melts from the 3rd up to the 8th generation were studied and a six order of magnitude jump in viscosity was found between the 5th and the 6th generations. In time, these results practically coincided with the modeling of carbosilane dendrimer melts [35,36], and somewhat later—with the experimental observation of some ordering in these systems [37]. Despite these important results, we are still far from understanding the mechanisms of dendrimer melt dynamics. 

From one point of view, a sharp increase in the viscosity of high-generation dendrimer melts could be associated with an increasing size of dendrimer molecules and the colloidal nature of the flow of these objects [38,39]. A serious argument against this mechanism was the result on the melt rheology of multi-arm polydimethylsiloxane (PDMS) stars, which were significantly superior to dendrimers in size and molecular weight [40]. Large dimensions, a dense globular shape, and a high molecular weight do not prevent them from maintaining the molecular type of flow, as evidenced by the low values of the melt viscosity and practically indistinguishable values of the activation energy of viscous flow in comparison with their linear PDMS analogs [40]. Another possible mechanism of an unprecedented jump in the viscosity of high-generation carbosilane dendrimer melts that have been proposed in Refs. [34,41] is the appearance of some topological entanglements of a new type, which are characteristic for high-generation dendrimers, owing to their denser molecular structure. However, no confirmation of any entanglement formation was found in the computer modeling of carbosilane dendrimer melts [36,42]. This means that further studies are necessary.

In this work, we continue our studies of the liquid–solid transition on a series of hybrid dendrimers, which can be formally imagined as carbosilane dendrimers with trimethylsiloxy groups that replace butyl ones at the silicon atom in the terminal layer. Such a replacement, carried out within the framework of the original synthetic scheme, made it possible to further compact the surface layer. We call these dendrimers as polycarbosilane-siloxane hybrid dendrimers because of their structure. Like pure polycarbosilane dendrimers, these hybrids also lack specific interactions, and it is expected that their bulk behavior is determined by the regular tree-like architecture of the dendrimers. Atomic models of dendrimers that were obtained by molecular dynamics were validated using small-angle X-ray scattering (SAXS)

## 2. Materials and Methods

### 2.1. Materials

Hexamethyldisilazane was purchased from Acros (Geel, Belgium) and used without further purification. Methyldichlorosilane was distilled directly before use. Toluene was distilled from Na/benzophenone, and hexane and pyridine were distilled from calcium hydride and barium oxide, respectively, prior to use. Other regular chemical reagents were used as received. The synthesis of polycarbosilane dendrimers G3 (Si_29_All_32_), G5 (Si_125_All_128_), and G6 (Si_253_All_256_) was carried out according to literature procedures [29,43,44] that were based on the cycle of reactions of organomagnesium synthesis using allyl chloride and magnesium metal, as well as hydrosilylation reaction using methyldichlorosilane and platinum catalyst (Sigma-Aldrich, Saint Louis, MO, USA)—a complex of zero-valent platinum with 1,3-divinyl-1,1,3,3-tetramethyldisiloxane (solution in xylene). The resulting carbosilane dendrimers were used in further conversions without further purification.

### 2.2. Methods

#### 2.2.1. Gel Permeation Chromatography (GPC)

GPC analysis was performed on a chromatographic system consisting of a high-pressure pump Stayer 2 (Aquilon, Moscow, Russia), a Smartline RI 2300 (KNAUER, Berlin, Germany) refractometer (using the tetrahydrofuran as an eluent), or a RIDK 102 refractometer (Prague, Czech Republic) (using toluene as an eluent) and a thermostat of JETSTREAM 2 PLUS columns (KNAUER, Berlin, Germany). The temperature of thermostating is 40 °C (±0.1 °C).

The eluents are tetrahydrofuran (THF) or toluene + 2% THF, the flow rate is 1.0 mL/min. Columns of 300 mm in length and 7.8 mm in diameter (300 × 7.8 mm^2^) are filled with Phenogel sorbent (Phenomenex, Los Angeles, CA, USA), particle size 5 μm, pore size 10^3^ Å and 10^4^ Å (passport range up to 75,000 D and up to 500,000 D, respectively). The registration and processing of data were carried out with the help of UniChrom 4.7 (Minsk, Belarus).

#### 2.2.2. Preparative Chromatography

The preparative chromatographic system consisted of a high-pressure isocratic pump LC-20AT (SHIMADZU, Kyoto, Japan), a RIDK-102 refractometric detector (Waters Corporation, Prague, Czech Republic), and 300 × 21.2 mm^2^ Phenomenex preparative columns (USA) that were packed with the Phenogel sorbent (particle size 10 µm). Columns with pore sizes of 10^3^ Å and 10^4^ Å were used and toluene was used as the eluent, depending on the molecular masses of the components of the mixtures being separated.

#### 2.2.3. Nuclear Magnetic Resonance Spectroscopy (NMR)

The NMR spectra were recorded on a Bruker Avance AV-300 spectrometer (300 MHz for ^1^H, 77.5 MHz for ^13^C, 59.6 MHz for ^29^Si), the internal standard was tetramethylsilane, and the solvent was CDCl_3_. The spectra were processed on a computer while using the ACD/ChemSketch, version 2020.1.2, Advanced Chemistry Development, Inc., Toronto, ON, Canada, www.acdlabs.com, 2021.

#### 2.2.4. Gas-liquid Chromatography (GLC) 

The GLC analysis was performed on a “Chromatech Analytic 5000” chromatograph (Russia) with katharometer as detector, helium as carrier gas, with 2 m × 3 mm column, and stationary phase SE-30 (5%) was applied to Chromaton-H-AW. Registration and data collection was carried out with the help of the program “Chromatech Analyst” (Yoshkar-Ola, Russia). 

#### 2.2.5. Differential Scanning Calorimetry (DSC)

Using the DSC method, the samples were examined on a differential scanning calorimeter “Mettler-822e” at a heating rate of 20 deg/min. in an argon atmosphere. The glass transition temperature was determined in the middle of the step on the DSC thermogram. 

### 2.3. Synthesis of Compounds

#### 2.3.1. Synthesis of the Trimethylsilanol

To the three-neck round-bottom flask containing 110 g (0.7 mmol) of hexamethyldisilazane (GMDS), 214 mL of a 0.1 N hydrochloric acid solution were added dropwise slowly through a dropping funnel with continuous stirring. The conversion of GMDS was checked several times during the adding of the acid. When the GMDS was completely exhausted, the organic solution of the reaction mixture was dried with anhydrous calcium chloride overnight. The content of trimethylsilanol in the final product was 95%, according to GLC. 

#### 2.3.2. Synthesis of the 1,1,1,3,5,5,5-heptamethyltrisiloxane

The methyldichlorosilane (20 g, 0.17 mol) dissolved in 20 mL of hexane was added to the two-neck round-bottom flask, containing the solution of trimethylsilanol (40 g, 0.45 mol) and pyridine (33.2 g, 0.42 mol) in 20 mL of hexane at 60 °C through a dropping funnel. After the end of the dropping, the reaction mixture was cooled and the solution reacted continually overnight under magnetic stirring at room temperature. After the filtration from the precipitate and washing with water for three times, the organic solution was dried with anhydrous sodium sulfate. The 19.3 g of resulting product was isolated by distillation at atmospheric pressure (bp = 142 °C), with the yield of 50%. ^1^H NMR (300 MHz, CDCl_3_, δ): 0.12 (s, 21H, Si-CH**_3_**), 4.64 (s, 1H, Si-H). ^29^Si NMR (59.6 MHz, CDCl_3_, δ): −36.43 Si-H, 9.35 Si(CH_3_)_3_. 

#### 2.3.3. Synthesis of the Carbosilane Dendrimer with the Heptamethyltrisiloxane Shell of the 4th, 6th and 7th Generation (G4(OTMS)_32_, G6(OTMS)_128_ and G7(OTMS)_256_)

To the one-neck round-bottom flask containing the solution of 1.5 g (0.4 mmol) of polycarbosilane dendrimer G3 (Si_29_All_32_) and 4.3 g (19.4 mmol) of 1,1,1,3,5,5,5-heptamethyltrisiloxane in 30 mL of toluene 30 µL of platinum catalyst were added. Subsequently, the solution reacted continually for 24 h under magnetic stirring at room temperature. Afterwards, the crude product was subjected to column chromatography on silica gel with toluene as the eluent to afford 3 g of the dendrimer with the substituted allyl groups with a yield of 70%. According to the GPC MM = 7000. ^1^H NMR (300 MHz, CDCl_3_, δ): −0.06 (s, 84H, Si-CH**_3_**), 0.00 (s, 96H, Si(CH**_3_**)(O-Si(CH_3_)_3_)_2_), 0.10 (s, 576H, Si(CH_3_)(O-Si(CH**_3_**)_3_)_2_), 0.44–0.7 (m, 232H, -CH**_2_**-Si-CH**_2_**-), 1.19–1.46 (m, 120H, Si-CH_2_-CH**_2_**-CH_2_-Si). ^29^Si NMR (59.6 MHz, CDCl_3_, δ): −21.86 Si(CH_3_)(O-Si(CH_3_)_3_)_2_, 1.05 Si-CH_2-_CH_2_-CH_2_-, 6.64 O-Si(CH_3_)_3_.

Carbosilane dendrimer with the heptamethyltrisiloxane shell of the six generation (G6(OTMS)_128_) was synthesized by changing the initial dendrimer to polycarbosilane dendrimer G5 (Si_125_All_128_) (1.27 g, 0.08 mmol) with the same procedures using 3.41 g (15.4 mmol) of 1,1,1,3,5,5,5-heptamethyltrisiloxane. The crude product was subjected to column chromatography on silica gel with toluene as the eluent to afford 2.5 g of the resulting dendrimer with a yield of 70%. According to the GPC MM = 16000. ^1^H NMR (300 MHz, CDCl_3_, δ): −0.08 (s, 372H, Si-CH**_3_**), −0.03 (s, 384H, Si(CH**_3_**)(O-Si(CH_3_)_3_)_2_),. 0.07 (s, 2304H, Si(CH_3_)(O-Si(CH**_3_**)_3_)_2_), 0.44–0.7 (m, 1008H, -CH**_2_**-Si-CH**_2_**-), 1.19–1.46 (m, 504H, Si-CH_2_-CH**_2_**-CH_2_-Si). ^29^Si NMR (59.6 MHz, CDCl_3_, δ): −21.96 Si(CH_3_)(O-Si(CH_3_)_3_)_2_, 1.03 Si-CH_2-_CH_2-_CH_2_-, 6.53 O-Si(CH_3_)_3_; Anal. Calcd C_1776_H_4572_O_256_Si_509_ (44232 g/mol): C, 48.18; Si, 32.22; H, 10.34; Obt.: C, 48.06; Si, 32.10; H, 10.21.

Carbosilane dendrimer with the heptamethyltrisiloxane shell of the seven generation (G7(OTMS)_256_) was synthesized by changing the initial dendrimer to polycarbosilane dendrimer G6 (Si_253_All_256_) (1.5 g, 0.047 mmol) with the same procedures utilizing 4.1 g (18.5 mmol) of 1,1,1,3,5,5,5-heptamethyltrisiloxane. The crude product was subjected to column chromatography on silica gel with toluene as the eluent to afford 2.9 g of the resulting dendrimer with a yield of 70%. According to the GPC MM = 19500. ^1^H NMR (300 MHz, CDCl_3_, δ): −0.06 (s, 756H, Si-CH**_3_**), 0.00 (s, 768H, Si(CH**_3_**)(O-Si(CH_3_)_3_)_2_),. 0.10 (s, 4608H, Si(CH_3_)(O-Si(CH**_3_**)_3_)_2_), 0.44–0.7 (m, 2032H, -CH**_2_**-Si-CH**_2_**-), 1.19–1.46 (m, 1016H, Si-CH_2_-CH**_2_**-CH_2_-Si). ^29^Si NMR (59.6 MHz, CDCl_3_, δ): −22.00 Si(CH_3_)(O-Si(CH_3_)_3_)_2_, 1.01 Si-CH_2-_CH_2-_CH_2_-, 6.49 O-Si(CH_3_)_3_; Anal. Calcd C_3568_H_9180_O_512_Si_1021_ (88776 g/mol): C, 48.23; Si, 32.20; H, 10.34; Obt.: C, 48.76; Si, 32.72; H, 10.41.

### 2.4. Rheological Studies

Rheological measurements were performed on the commercially available rheometer Anton Paar, the model Physica MCR-302. The flow curves of G4(OTMS) were carried out under steady shear with the use of a 50 mm plate-plate measuring unit at the temperature of 20 °C, 40 °C, 60 °C, and 80 °C. Temperature control was achieved with a Peltier plate. The viscosity measurements of G6(OTMS) and G7(OTMS) were performed via creep experiments. The samples for the measurements were prepared according to the following procedure: a dendrimer sample was fixed in a mold and then pressed into a disk with the diameter of 30 mm and the thickness of 0.2–0.3 mm at 25 °C, and then held under the pressure of 10 MPa for 10 min. Disks with the diameter of 25 mm were cut out from the obtained samples for the rheological measurements with the use of a plate-plate measuring units of 25 mm in diameter. The time dependences of the creep compliance were obtained and the viscosity values were found from fitting by Burgers model.

### 2.5. Molecular Dynamics Simulations

Atomic models of the G4 (OTMS), G6 (OTMS) and G7 (OTMS) were generated using the PUMA software package [45,46]. The models were fully atomistic, except for the ethylene and methyl groups, being treated as united atoms. The potentials accounting for bond stretching and bond bending were introduced in the same functional form with the parameters from the AMBER [47,48] force field for the carbosilane part and PCFF [49] for the siloxane part of the hybrid dendrimers. The torsion angle potential was taken into account for the carbosilane monomer units. Non-bonded interactions of all atoms were modeled using the Lenard–Jones (LJ) 6–12 potential. Besides, Coulomb interactions of partial charges were also taken into account via the screened Coulomb potential. The cut-off distance was 10.5 Å for both LJ and Coulomb interactions. Tables S1–S4 presents all the parameters of the force fields used in this work.

Equilibrated conformations of each dendrimer were obtained at the temperature of 300 K. Standard MD techniques with the collisional thermostat [50,51] have been used for the dendrimer relaxation. The elementary integration step was 0.001 ps, and the total equilibration time of all dendrimerы was 1 ns; the simulation time of one run reached 6 ns. Achieving steady state was controlled by monitoring all of the contributions to the energy of the system. 

### 2.6. Solution Scattering Experiments and Data Analysis

Synchrotron SAXS measurements of the G4 (OTMS), G6 (OTMS) and G7 (OTMS) were performed at the European Molecular Biology Laboratory (EMBL) on the P12 BioSAXS beam line at the PETRA-III storage ring (DESY, Hamburg) that was equipped with a robotic sample changer and a 2D photon counting pixel X-ray detector Pilatus 2M (DECTRIS, Switzerland). The scattering intensity, *I*(*s*), was recorded in the range of the momentum transfer 0.03 < *s* < 7.3 nm^−1^, where *s* = (4πsinθ)/λ, 2θ is the scattering angle, and λ = 0.124 nm, the X-ray wavelength [52]. The measurements were carried out using continuous sample flow operation over a total exposure time of 1 s, collected as 20 × 50 ms individual frames to monitor for potential radiation damage (no radiation effects were detected [53]). The data were corrected for the solvent scattering and processed while using standard procedures with the program suite ATSAS [54]. The concentration series from 1 to 25 mg/mL were measured and the corresponding scattering profiles were merged to account for the interparticle interactions.

The radii of gyration *R_g_* were evaluated using the Guinier approximation [55] assuming that, at very small angles (*s* < 1.3/*R_g_*), the intensity is represented as *I*(*s*) *= I*(0) *exp*(−1/3(*sR_g_*)^2^) and the volumes of the hydrated particles *V_p_* were estimated using the Porod invariant [56]. The maximum dimension *D_max_* was computed using the indirect transform package GNOM [57], which also provides the distance distribution function *p*(*r*). The scattering from the atomic models that was generated by molecular dynamics was calculated using CRYSOL [58], which fits the experimental profile by adjustment of the total excluded volume of the particle inaccessible to the solvent.

## 3. Results and Discussion

The main objective of the present study is to create prerequisites for the synthesis and systematic study of polycarbosilane dendrimers with a siloxane shell, which is characterized by the absence of specific interactions, such as hydrogen bonds and electrostatic interactions. 

### 3.1. Synthesis of Dendrimers

**Figure 1:**
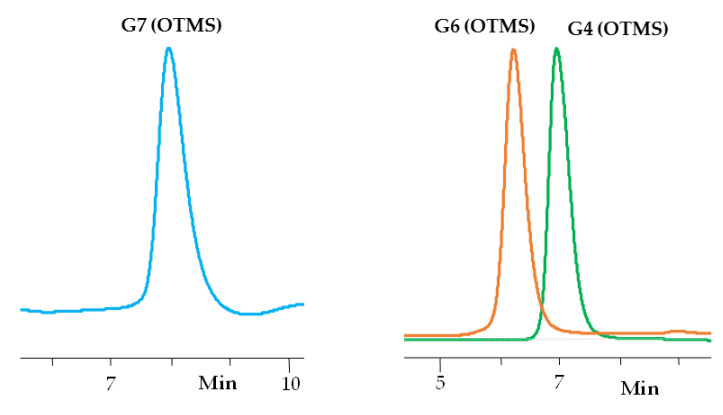
Gel Permeation Chromatography (GPC) curves of the obtained carbosilane-siloxane dendrimers (MSOCSD G4 (OTMS), and G6 (OTMS), G7 (OTMS)) after chromatographic purification.

The polymer matrices for the creation of the target compounds were carbosilane dendrimers of the 3rd, 5th, and 6th generations with a four-functional branching center and a surface layer consisting of diallylmethylsilyl groups, and being obtained via layer-by-layer synthesis according to the previously improved method [34]. This method allows for one to obtain these compounds with a purity of ~ 93% according to the data of gel permeation chromatography (Figures S4, S7 and S10 in Supplementary Materials). The presence of the outer layer consisting of diallylmethylsilyl groups made it possible to modify it using the hydrosilylation reaction [59]. To create a siloxane shell, we used a monofunctional 1,1,1,3,5,5,5-heptamethyltrisiloxane containing a hydridsilyl functional group at the central silicon atom. The synthesis of such a monofunctional trisiloxane was carried out according to the procedure described in Ref. [60]. The hydrosilylation reaction was carried out in the presence of a Carsted catalyst at room temperature. All of the resulting dendrimers were purified by preparative chromatography to obtain pure products, denoted as G4(OTMS), G5(OTMS), and G7(OTMS) (with the number indicated the dendrimer generation) (Figure 1). The structure of the obtained dendrimers was also confirmed by elemental analysis data.

**Figure 2:**
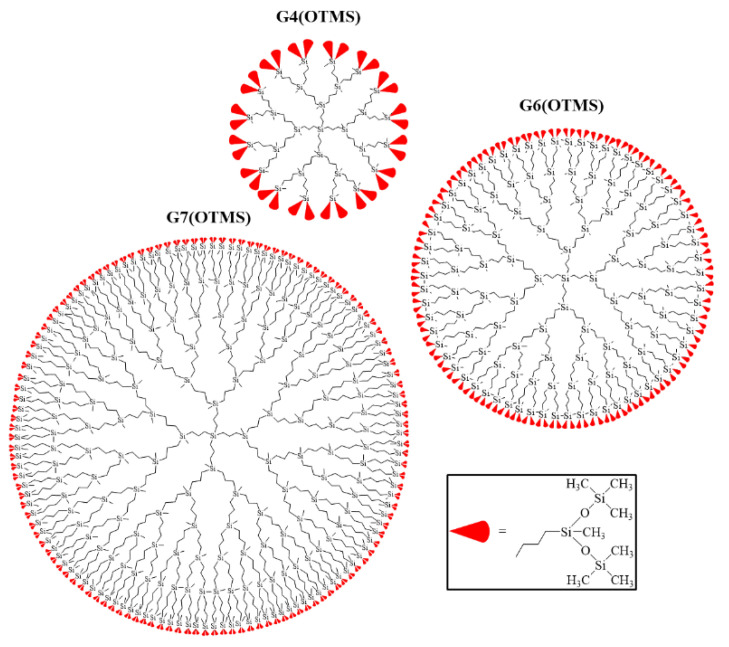
Homologous series of the obtained carbosilane dendrimers G4 (OTMS), G6 (OTMS), and G7 (OTMS) with a non-functional siloxane shell schematically shown by red.

Figure 2 schematically depicts the structure of the synthesized homologous series of carbosilane-siloxane dendrimers with a non-functional siloxane terminal layer. The preparation and subsequent purification of the obtained samples allowed for us to investigate some physical properties of the obtained dendrimers using three generations as examples and, thus, lay the foundations for further detailed studies of the structure-property relationship for a given hybrid dendrimer species.

### 3.2. Thermophysical Properties

A number of physicochemical methods of analysis were used to elucidate the effect of the peripheral shell structure of the carbosilane dendrimer on the resulting properties of the obtained compounds.

**Figure 3:**
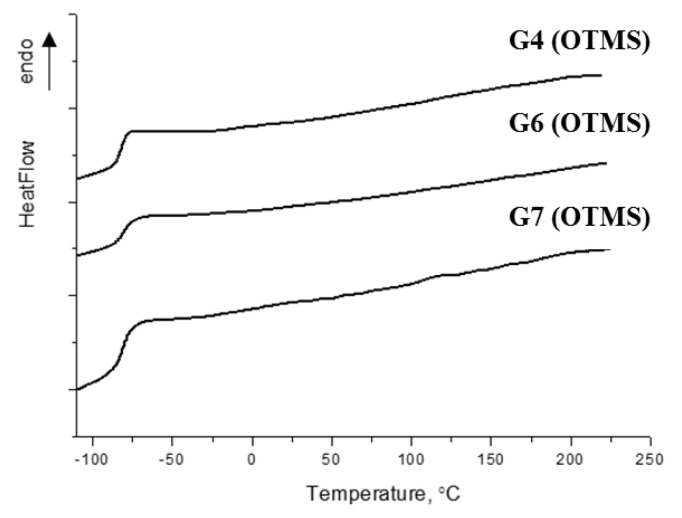
Thermograms for the 4th, 6th, and 7th generations of hybrid carbosilane-siloxane dendrimers (G4 (OTMS), G6 (OTMS), and G7 (OTMS)) in an argon atmosphere at a heating rate of 20 °C/min.

The glass transition temperature was measured by differential scanning calorimetry. Figure 3. shows thermograms for the 4th, 6th, and 7th generations of carbosilane-siloxane dendrimers in an argon atmosphere at a heating rate of 20 °C/min.

The thermograms display a jump in the heat flow in the range from −90 °C (183 K) to −75 °C (198 K) for all three generations, corresponding to the devitrification interval, which defines the glass transition temperature *T*_g_ = −83 °C as being the same for all dendrimers under study. This result confirms the conclusion that *T*_g_ is independent of generation for high-generation dendrimers which was reported in Ref. [33]. On the other hand, it has previously been shown that the nature of the terminal groups has a decisive influence on the mobility of dendrimer macromolecules and, accordingly, on *T*_g_ [61,62].

Indeed, the analysis of the available literature data demonstrated a high sensitivity of the mobility of dendrimer molecules to the structure of the outer layer. Namely, in spite of the general dimethylsiloxane nature of the terminal groups of the third generation carbosilane dendrimers, the *T*_g_ of a number of samples changed with a change in its architecture (Table 1). An increase in the mobility of a terminal group on going from a linear dimethylsiloxane chain to dimethylcyclotetrasiloxane led to an increase in *T*_g_, while the samples that were synthesized in this work (Table 1, item 4) occupied an intermediate position. At the same time, for the 3rd generation, a change in the nature of the carbosilane dendrimer (Table 1, item 4) to a more mobile siloxane (Table 1, item 6) with similar trimethylsilyl end groups led to a decrease in *T*_g_. For high-generation carbosilane dendrimers with butyl terminal groups *T*_g_ = −87 °C [33], this value is lower than *T*_g_ = −83 °C that was found for the hybrid carbosilane-siloxane dendrimers.

### 3.3. Rheological Behavior

Table 2 presents the values of the intrinsic viscosity obtained for all synthesized dendrimers in toluene. One can see that [η] hardly depends on the dendrimer generation number. The similar conclusion was drawn for carbosilane homologues series [7]. However, the value of [η] is smaller for carbosilane-siloxane hybrids that seem to have a denser molecular structure due to the substitution of -CH_3_ terminal groups in carbosilane dendrimers with more massive -SiMe[OSiMe_3_]_2_ segments.

The rheology of the synthesized compounds in bulk is of particular interest. It has been mentioned above that a transition from a liquid-like to a solid-like behavior was found for non-functional carbosilane dendrimers with butyl-terminated groups upon increasing dendrimer generation [29,30,31,32,33,34]. This transition was accompanied by a very strong jump-like rise in melt viscosity [34]. At the same time, the storage modulus of dendrimer melts became much higher than the loss modulus G’’, starting from the 6th generation. It was suggested that such a unique change in viscoelastic properties of dendrimer melts is associated with the specific regular tree-like architecture of dendrimers and it could be related to the formation of a spatial physical network by neighboring dendrimer molecules via the penetration of peripheral groups into the surface layer of neighboring macromolecules [29,30]. It could be assumed that a change in the nature of the peripheral layer of the dendrimer molecules would have an impact on this alleged process. However, the replacement of the rather rigid methyldibutylsilyl terminal groups by the more mobile siloxane ones in our case did not change the general trends in viscoelastic behavior of dendrimer melts, but caused just some slight shift in the viscosity values.

**Figure 4:**
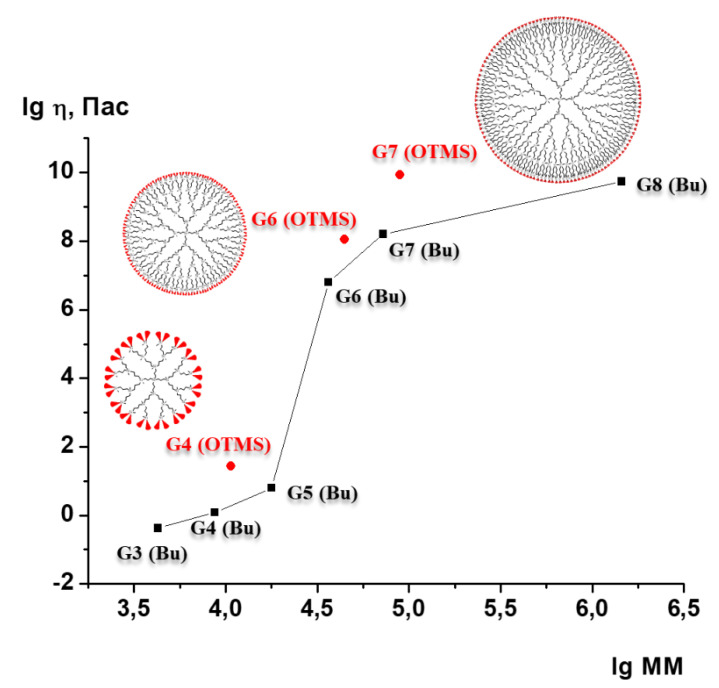
Viscosity of the hybrid polycarbosilane-siloxane dendrimers versus their molecular mass (red circles) compared to the MM dependence of the viscosity of the homologous series of poly(bytil)carbosilane dendrimers (black squares) studied earlier [34].

Figure 4 demonstrates the dependence of the melt viscosity on MM of the hybrid dendrimers in comparison with that for carbosilane dendrimers obtained in [34]. One can see that the trimethylsilylsiloxane peripheral layer led to an increase in melt viscosity of dendrimers of all generations (together with *T*_g_), but it did not change the main trend. Like pure carbosilane G4 dendrimers, the G4(OTMS) is a Newtonian fluid (Figure S22 the in Supplementary Materials show its flow curves at various temperatures) with a rather low viscosity value and the activation energy of viscous flow equal 30 kJ/mol, while the viscosities of G6(OTMS) and G7(OTMS) are more than six orders of magnitude higher. One can see that the MM dependence of the viscosity is qualitatively similar to that found for carbosilane dendrimers. Actually, the G6(OTMS) and G7(OTMS) lose their ability to freely flow acquiring some plasticity that is characterized by the existence of a yield stress. The flow of these materials was realized at low shear rates (10^−4^–10^−6^ s^−1^) in the creep regime similarly to G6–G8 carbosilane dendrimers [34]. The viscosity values of G6(OTMS) and G7(OTMS) that are shown in Figure 4 were obtained from the fitting of the creep curves with Burgers equation [65] (Figure S24 in Supplementary Materials). Thus, the hybrids also demonstrate a tremendous effect of the dendrimer generation on their bulk rheological properties and a transition from a Newtonian behavior of low-generation dendrimers to plastic solids that are realized with high-generation dendrimers.

### 3.4. Structural Modelling

**Figure 5:**
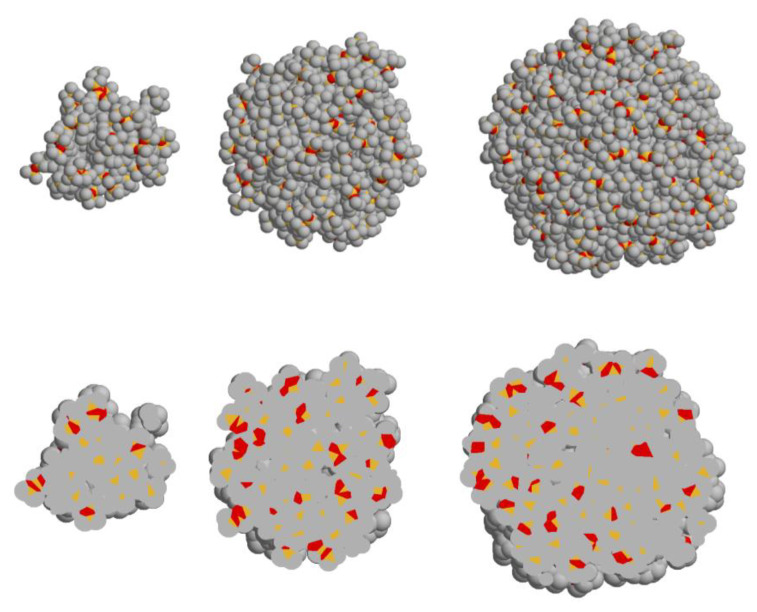
Molecular dynamics models of the 4th, 6th, and 7th generations of carbosilane-siloxane dendrimers. Top row: space-filling mode, bottom row: slab mode.

Atomic models of the 4th, 6th, and 7th generations of carbosilane-siloxane dendrimers were generated and evaluated via molecular dynamics (MD) simulations. The models were fully atomistic, except for the ethylene and methyl groups, being treated as united atoms. Figure 5 (upper row) shows the models in the space-filling mode. In this mode, each atom is represented as a sphere with a radius equal to its van der Waals radius. The snapshots of the dendrimer conformations that are represented in the slab mode (Figure 5, bottom row) allow for one to evaluate the surface layer of dendrimer molecules. Different colors are used for different types of atoms: silicon atoms are colored in yellow, oxygen atoms are shown by red, while gray is used to depict ethylene and methylene united atoms. It can be directly seen that with increasing generation, the sphericity of dendrimers increases. Mainly terminal methyl groups are located at the dendrimer periphery. The developed models were further used for fitting the SAXS data presented below.

### 3.5. X-ray Scattering Investigation

**Figure 6:**
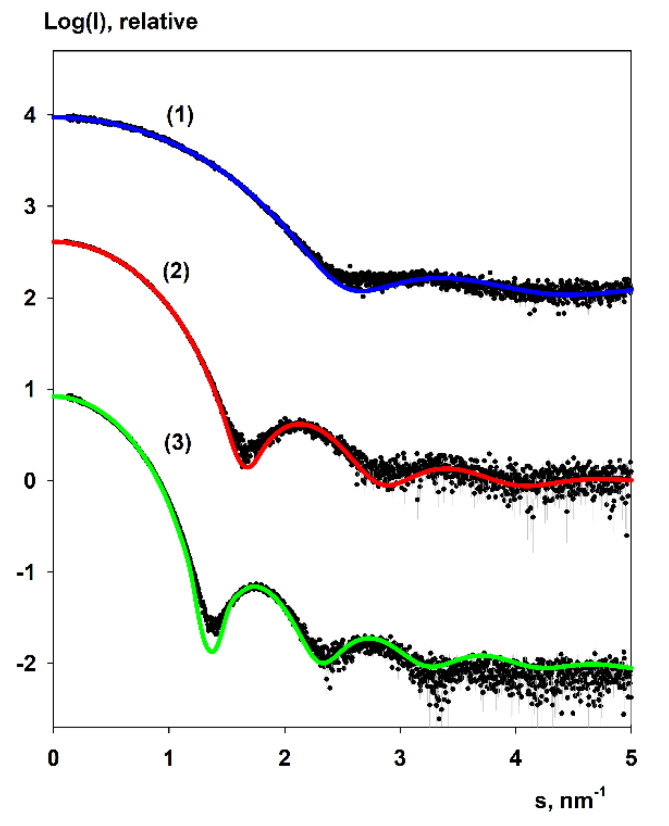
Small angle X-ray scattering (SAXS) patterns of the 4th (1), 6th (2), and 7th (3) generations of carbosilane-siloxane dendrimers. The experimental data are shown as dots with error bars, scattering from the MD models as solid lines.

**Figure 7:**
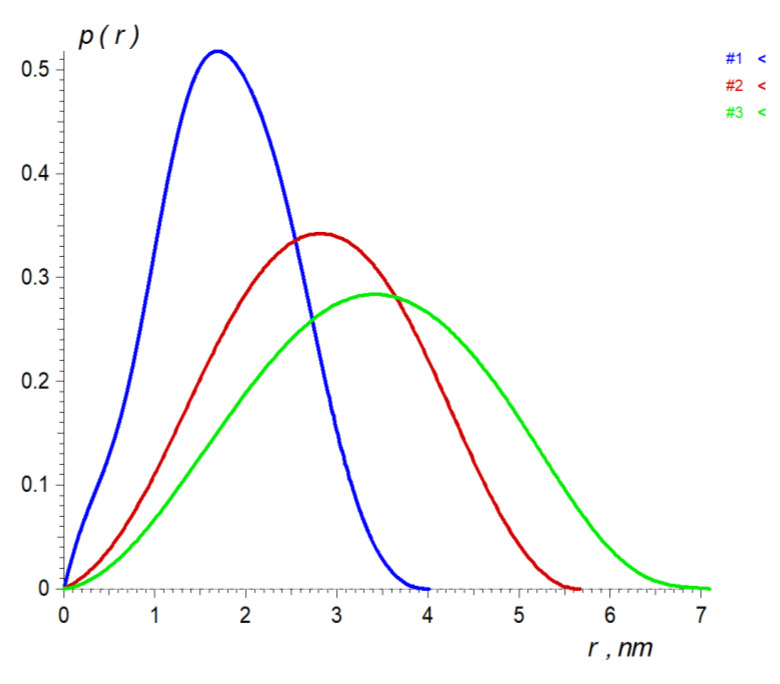
Distance distribution p(r) functions computed from the experimental SAXS data from the 4th (blue), 6th (red) and 7th (green) generations of carbosilane-siloxane dendrimers.

Small angle X-ray scattering (SAXS) has been applied to determine the overall structure of dendrimers and validate the generated MD models. Figure 6 presents the processed experimental scattering patterns and Table 3 gives the overall structural parameters computed from the SAXS data. The combination of the radii of gyration *R_g_* and the maximum size of the particles *D_max_* as well as the bell shapes of distance distribution functions *p*(*r*) (Figure 7) point to globular conformation of the particles in the solution.

The scattering from the MD models was evaluated by the program CRYSOL [58]. The program utilizes a spherical harmonics approach for the rapid calculation of the scattering amplitudes and isotropic SAXS intensities from high-resolution atomic structures of macromolecules and optionally fitting the calculated scattering to experimental SAXS data. The scattering intensity is attributed by three terms: the scattering of atomic structure in vacuum, scattering from excluded volume inaccessible to solvent, and optionally from the hydration shell with an electron density higher than that of the bulk solvent. During the fitting, the values of the total excluded volume of the particle, the average atomic group radius, and the contrast of the solvation shell are adjusted to minimize the discrepancy between the computed curve and experimental data. 

The theoretical scattering patterns computed from the molecular dynamics models of G4 (OTMS), G6 (OTMS), and G7 (OTMS) yield a fair agreement (Figure 6) with experimental scattering from the corresponding dendrimer. From this, we concluded the dendrimers are monodisperse and that their overall shapes are similar to those of the MD models.

## 4. Conclusions

In this work, hybrid carbosilane-siloxane dendrimers of three generations (G4, G6, and G7) were synthesized for the first time and characterized by several experimental methods. A comparative analysis of a number of physical properties of these dendrimers with the properties of pure carbosilane dendrimers, as well as dendrimers with some other end groups, made it possible to deepen our understanding of the influence of peripheral layers and the generation numbers on the conformational behavior and intermolecular interactions of carbosilane dendrimers. In particular, it was confirmed that Tg of high-generation dendrimers hardly depends on the generation number, but it is strongly affected by the chemical nature of the peripheral layer. Fitting of the SAXS data on dilute solutions of hybrid dendrimers with MD simulation models of these dendrimers showed a good agreement between the experimental and simulation results and demonstrated a monodispersity of dendrimers and their almost spherical shape.

The main significance of this work is the confirmation of the presence of a specific interaction between high-generation dendrimer molecules in bulk, which manifests itself in a jump of the melt viscosity with an increasing dendrimer generation and an effective liquid–solid transition. This effect was previously found for butyl-terminated carbosilane dendrimers and it is now obtained on carbosilane dendrimers with trimethylsiloxysilyl end groups. It is important that such a significant change in the nature and, accordingly, the mobility, of the surface layer did not change qualitatively rheological properties of these objects. This indicates the general nature of this phenomenon, which requires further in-depth study, since, apparently, it is the key to understanding the nature of dendrimers as a material with unusual properties. 

The generalization of previously obtained and new data on the relationship between the structure and properties of carbosilane dendrimers are important for the design of new materials, where the unique rheological behavior of these objects will be the decisive factor. 

## Figures and Tables

**Table 1:** Glass transition temperature of carbosilane dendrimers of the 3rd generation with terminal groups of different chemical nature.

Type of Terminal Groups	*T*_g_, °C	Ref.
-SiBu	−94	[29]
-SiAll_2_	−100	[63]
-SiMe-[OSiMe_2_]_4_-OSiMe_3_	−110	[30]
-SiMe[OSiMe_3_]_2_	−83	
-cyclotetrasiloxane	−66	[64]
methylsilsesquioxane dendrimer with terminal -OSiMe_3_	−102	[32]

**Table 2:** Intrinsic viscosity of the hybrid carbosilane-siloxane dendrimers.

Dendrimer	Intrinsic Viscosity Value [η], dL/g
G4(OTMS)	0.0256
G6(OTMS	0.0267
G7(OTMS)	0.0281

**Table 3:** Overall structural parameters obtained by SAXS.

Sample	*R_g_*, nm	*D_max_*, nm	*V_p_*, nm^3^
G4 (OTMS)	1.35	4.0	17.7
G6 (OTMS)	2.10	5.7	64.5
G7 (OTMS)	2.60	7.1	117.5

*R_g_* is the radius of gyration, *D_max_* is the maximum size, and *V_p_* is the excluded volume of the particle.

## Data Availability

The data presented in this study are available on request from the corresponding author.

## Supplementary Materials

The following are available online at https://www.mdpi.com/2073-4360/13/4/606/s1, Figure S1: ^1^H NMR spectrum of 1,1,1,3,5,5,5-heptamethyltrisiloxane, Figure S2: ^29^Si NMR spectrum of 1,1,1,3,5,5,5-heptamethyltrisiloxane, Figure S3: ^1^H NMR spectrum of the 3d generation of poly(allyl)carbosilane dendrimer (G3(All)), Figure S4: GPC curve of the 3d generation of poly(allyl)carbosilane dendrimer (G3(All)), Figure S5: ^1^H NMR spectrum of the product of hydrosilylation reaction of the 3d generation of poly(allyl)carbosilane dendrimer with 1,1,1,3,5,5,5-heptamethyltrisiloxane (G4(OTMS)), Figure S6: ^29^Si NMR spectrum of the product of hydrosilylation reaction of the 3d generation of poly(allyl)carbosilane dendrimer with 1,1,1,3,5,5,5-heptamethyltrisiloxane (G4(OTMS)), Figure S7: GPC curve of the 3d generation of poly(allyl)carbosilane dendrimer after the hydrosilylation reaction with 1,1,1,3,5,5,5-heptamethyltrisiloxane. Figure S8: GPC curve of the 4th generation of carbosilane-siloxane dendrimer (G4(OTMS)) after preparative chromatography purification, Figure S9: ^1^H NMR spectrum of the 5th generation of poly(allyl)carbosilane dendrimer (G5(All)), Figure S10: GPC curve of the 5th generation of poly(allyl)carbosilane dendrimer (G5(All)), Figure S11: ^1^H NMR spectrum of the product of hydrosilylation reaction of the 5th generation of poly(allyl)carbosilane dendrimer with 1,1,1,3,5,5,5-heptamethyltrisiloxane (G6(OTMS)), Figure S12: ^29^Si NMR spectrum of the product of hydrosilylation reaction of the 5th generation of poly(allyl)carbosilane dendrimer with 1,1,1,3,5,5,5-heptamethyltrisiloxane (G6(OTMS)), Figure S13: GPC curve of the 5th generation of poly(allyl)carbosilane dendrimer after the hydrosilylation reaction with 1,1,1,3,5,5,5-heptamethyltrisiloxane, Figure S14: GPC curve of the 6th generation of carbosilane-siloxane dendrimer (G6(OTMS)) after preparative chromatography purification, Figure S15: ^1^H NMR spectrum of the 6th generation of poly(allyl)carbosilane dendrimer (G6(All)), Figure S16: GPC curve of the 6th generation of poly(allyl)carbosilane dendrimer (G6(All)), Figure S17: ^1^H NMR spectrum of the product of hydrosilylation reaction of the 6th generation of poly(allyl)carbosilane dendrimer with 1,1,1,3,5,5,5-heptamethyltrisiloxane (G7(OTMS)), Figure S18: ^29^Si NMR spectrum of the product of hydrosilylation reaction of the 6th generation of poly(allyl)carbosilane dendrimer with 1,1,1,3,5,5,5-heptamethyltrisiloxane (G7(OTMS)), Figure S19: GPC curve of the 6th generation of poly(allyl)carbosilane dendrimer after the hydrosilylation reaction with 1,1,1,3,5,5,5-heptamethyltrisiloxane, Figure S20: GPC curve of the 7th generation of carbosilane-siloxane dendrimer (G7(OTMS)) after preparative chromatography purification, Figure S21: GPC curves of the 4th, 6th and 7th generations of carbosilane-siloxane dendrimers (G4(OTMS), G6(OTMS), G7(OTMS)) after preparative chromatography purification, Table S1: Bond potential, Table S2: Valence angle potential, Table S3: Torsion angle potential, Table S4: Lennard-Jones potential, Table S5: Atomic masses and partial charges of the atoms.
