# Supplementary material for: Hybrid Polycarbosilane-Siloxane Dendrimers: Synthesis and Properties
Source: Polymers (Basel). 2021 Feb 17;13(4):606. doi: 10.3390/polym13040606 (PMC7922192; doi:10.3390/polym13040606)
Supplement: Supplementary file 1 [file polymers-13-00606-s001.pdf]

polymers

MDPI

Supplementary Materials: Hybrid Polycarbosilane-Siloxane Dendrimers: Synthesis and Properties

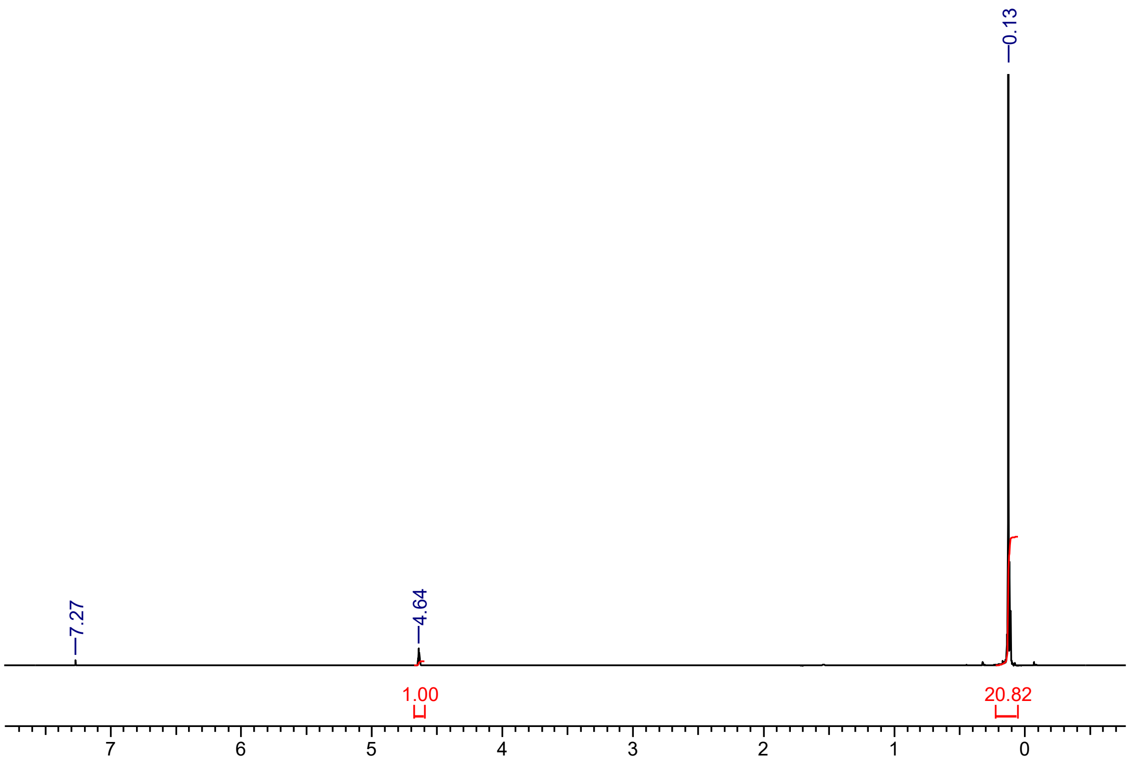

Figure S1. ^1H NMR spectrum of 1,1,1,3,5,5,5-heptamethyltrisiloxane.

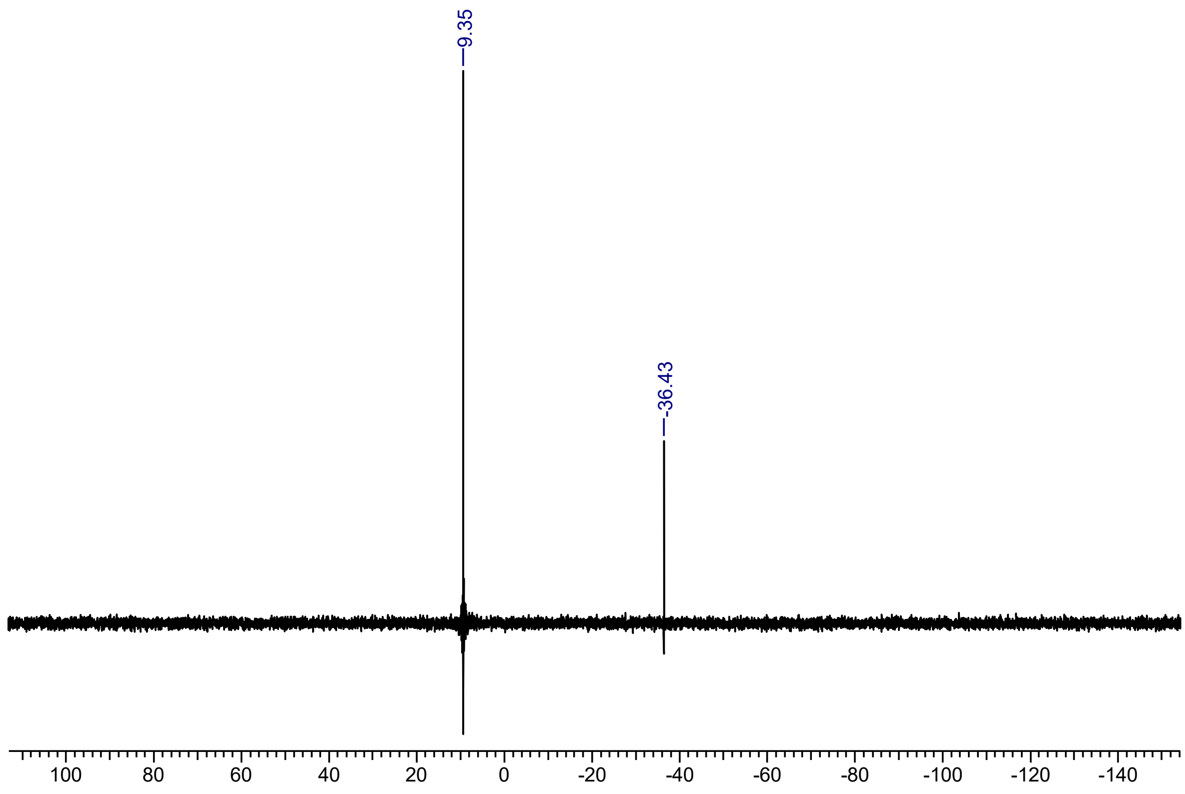

Figure S2. ^{29}Si NMR spectrum of 1,1,1,3,5,5,5-heptamethyltrisiloxane.

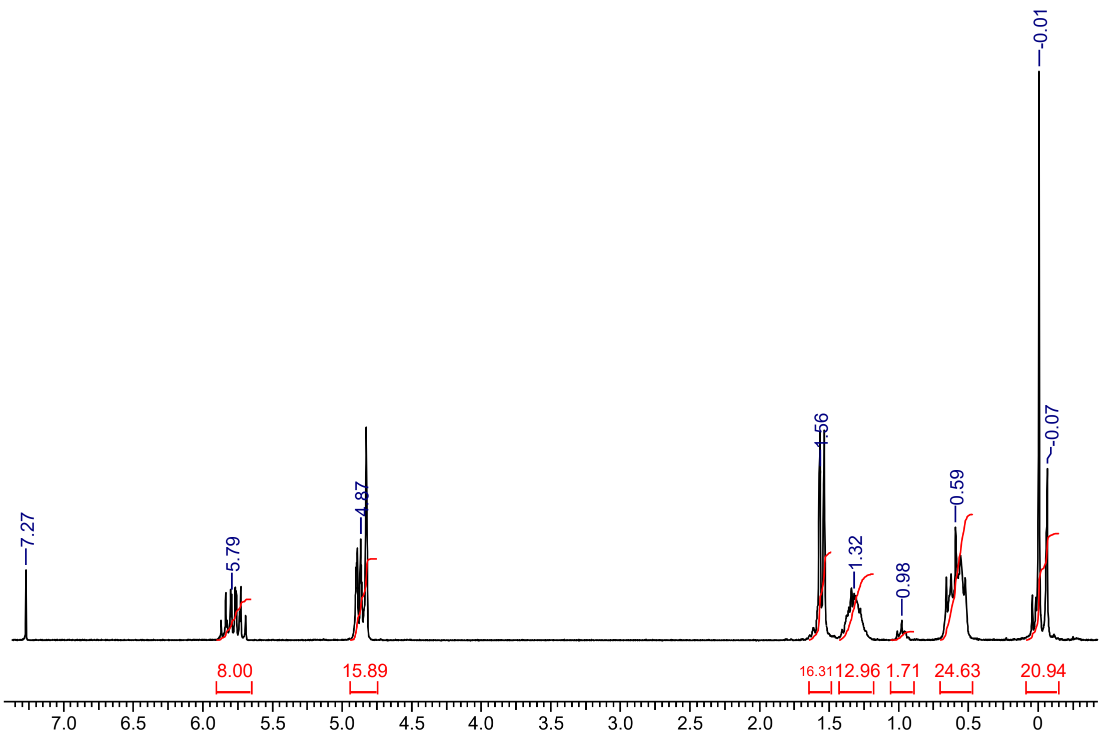

Figure S3. ^{1}H NMR spectrum of the 3rd generation of poly(allyl)carbosilane dendrimer (G3(All)).

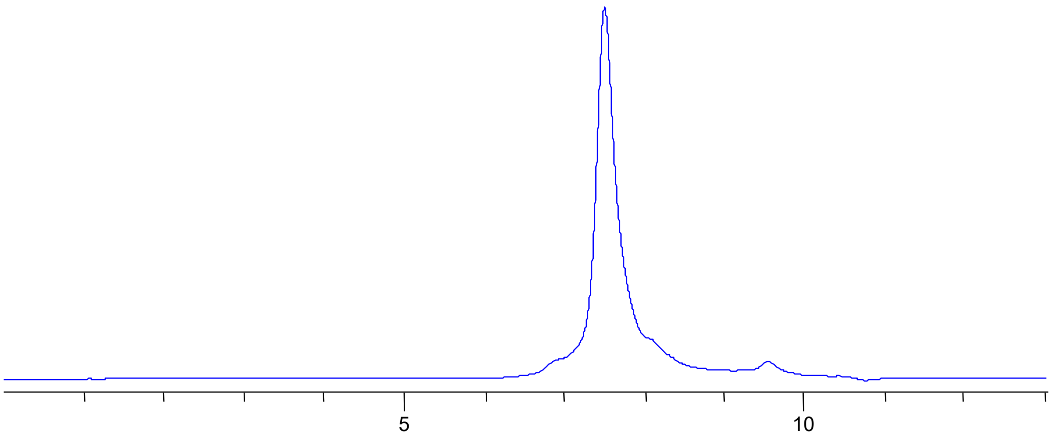

Figure S4. GPC curve of the 3rd generation of poly(allyl)carbosilane dendrimer (G3(All)).

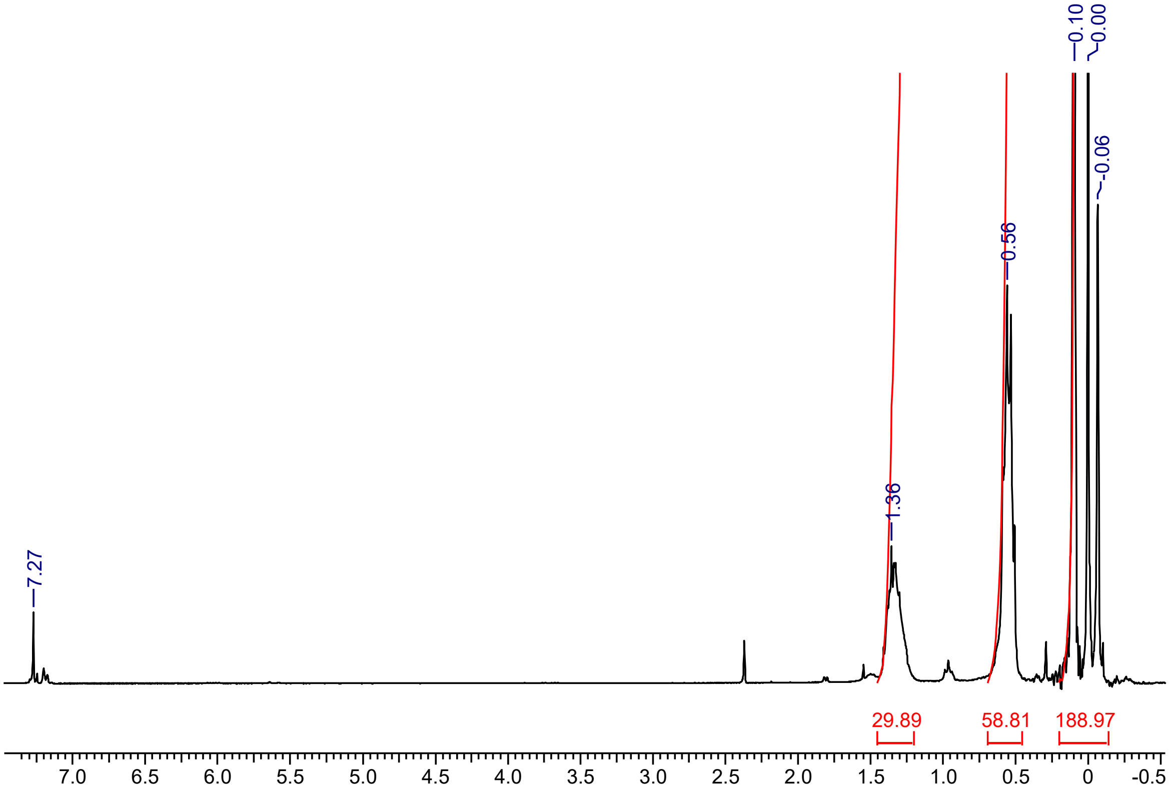

Figure S5. ^{1}H NMR spectrum of the product of hydrosilylation reaction of the 3rd generation of poly(allyl)carbosilane dendrimer with 1,1,1,3,5,5,5-heptamethyltrisiloxane (G4(OTMS)).

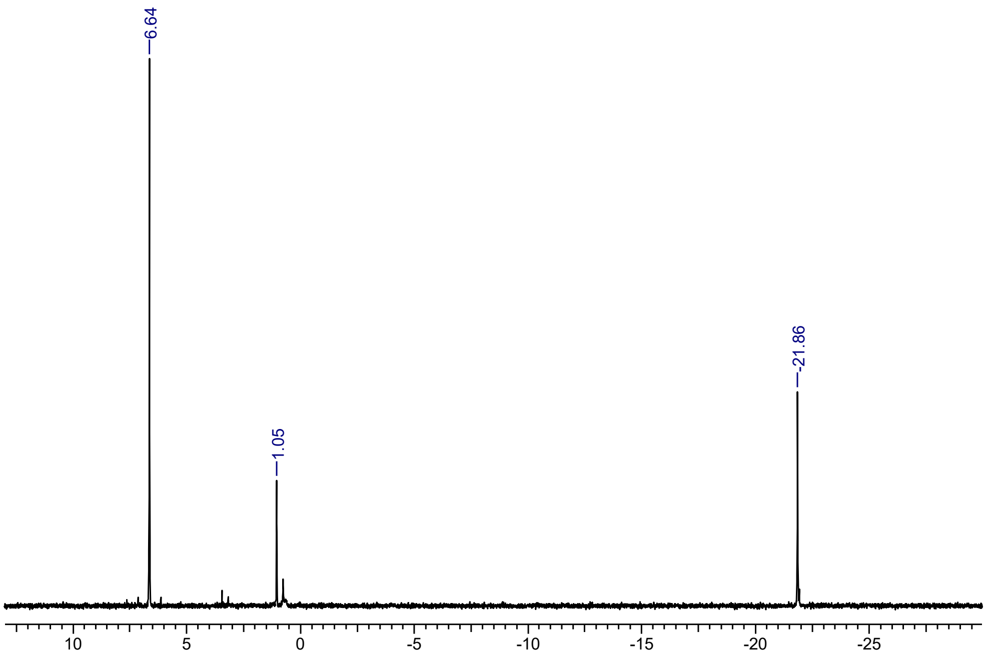

Figure S6. ^{29}Si NMR spectrum of the product of hydrosilylation reaction of the 3rd generation of poly(allyl)carbosilane dendrimer with 1,1,1,3,5,5,5-heptamethyltrisiloxane (G4(OTMS)).

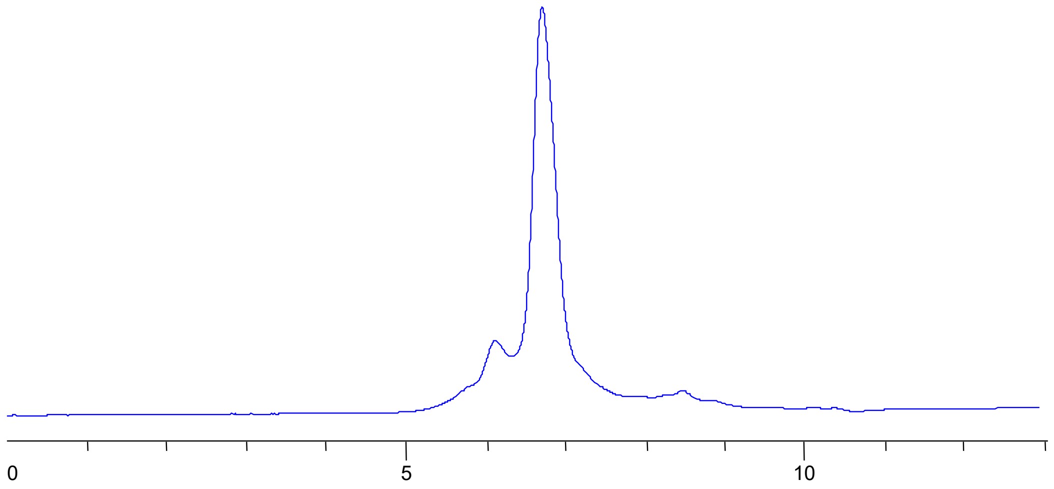

Figure S7. GPC curve of the 3rd generation of poly(allyl)carbosilane dendrimer after the hydrosilylation reaction with 1,1,1,3,5,5,5-heptamethyltrisiloxane.

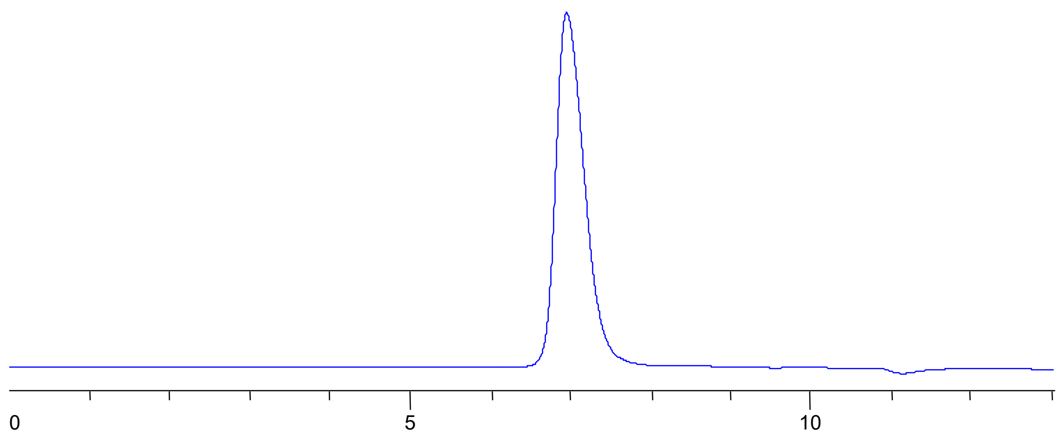

Figure S8. GPC curve of the 4th generation of carbosilane-siloxane dendrimer (G4(OTMS)) after preparative chromatography purification.

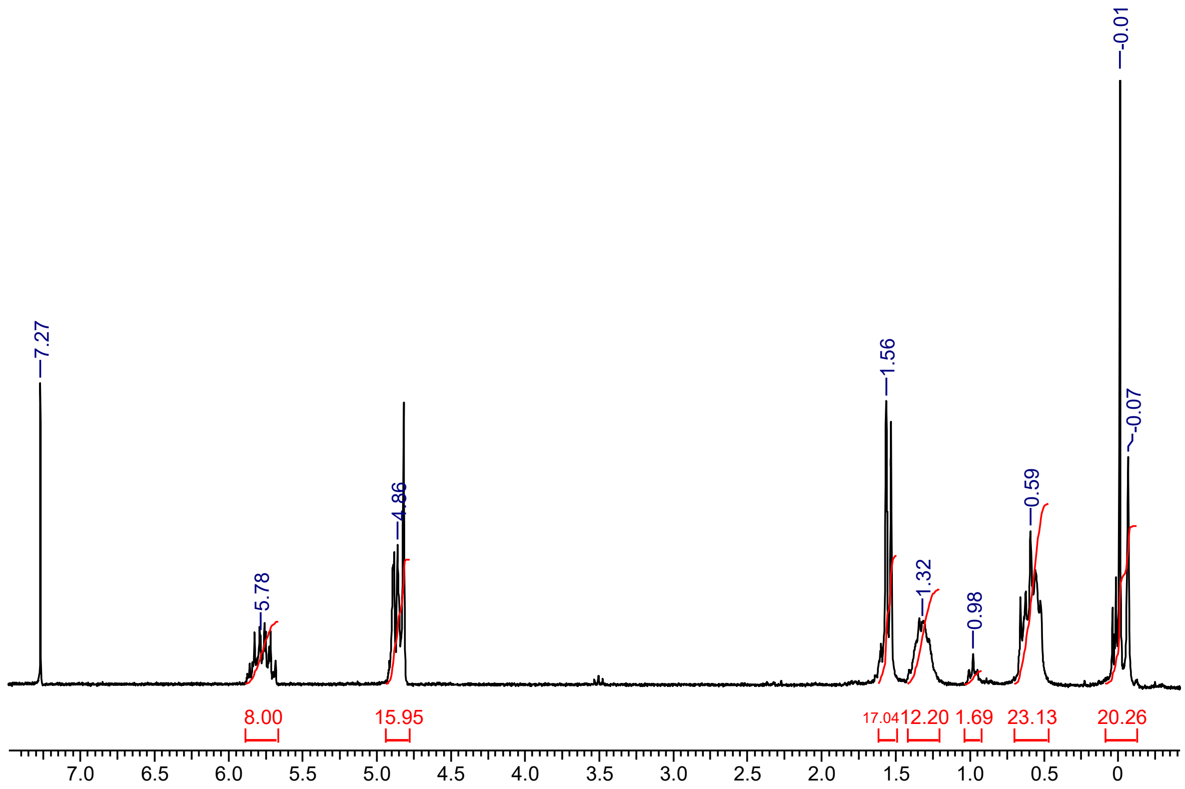

Figure S9. ^{1}H NMR spectrum of the 5th generation of poly(allyl)carbosilane dendrimer (G5(All)).

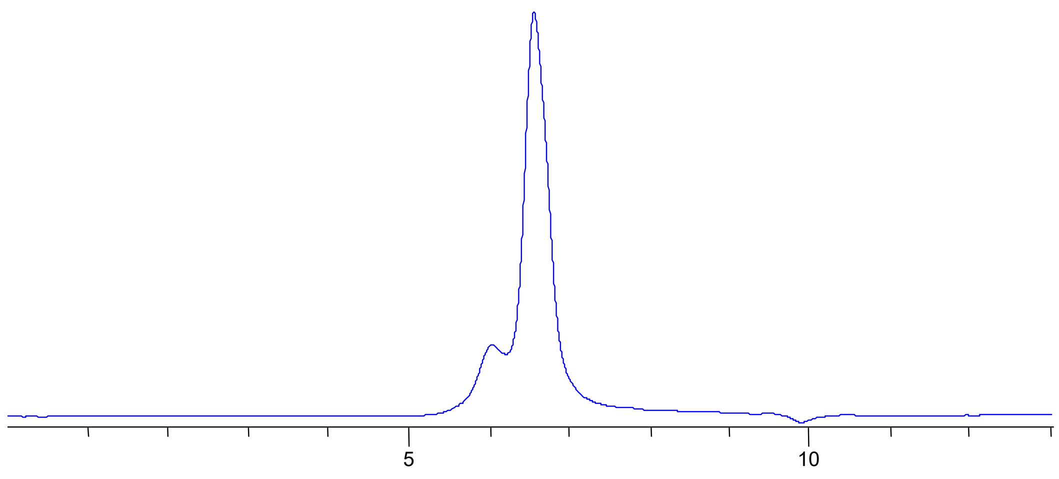

Figure S10. GPC curve of the 5th generation of poly(allyl)carbosilane dendrimer (G5(All)).

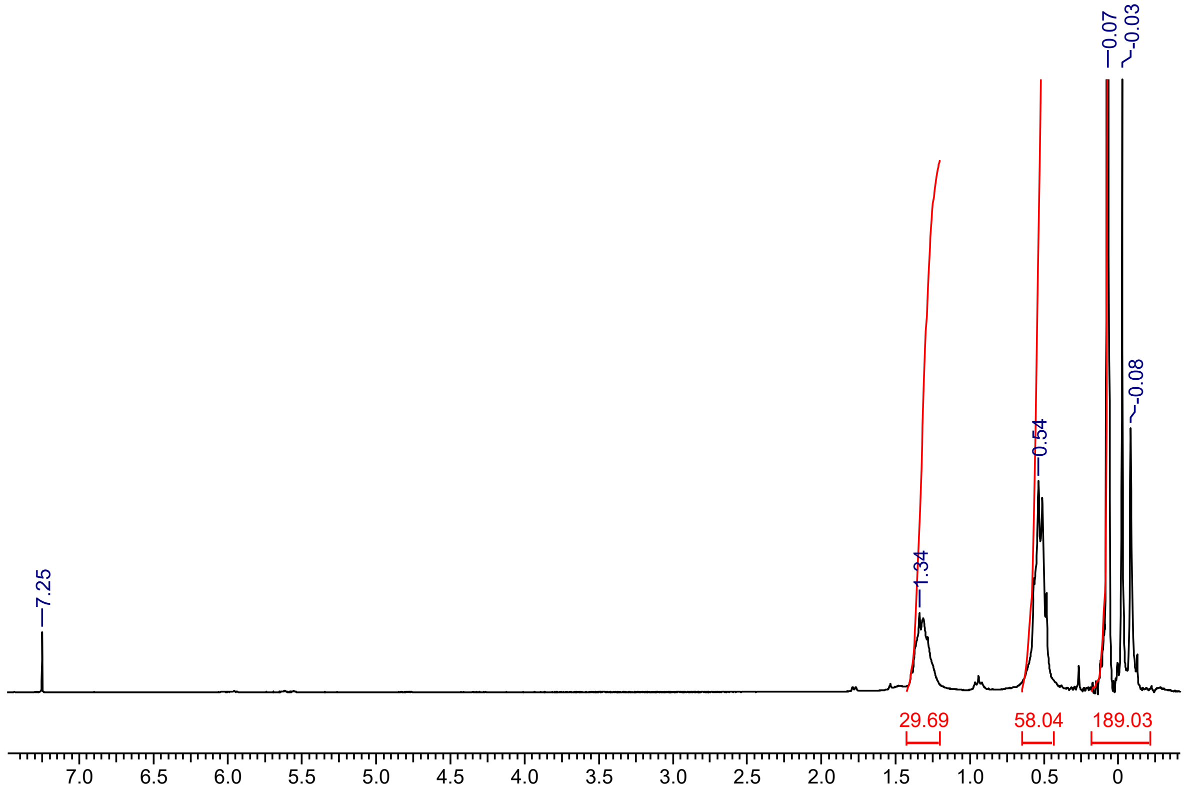

Figure S11. ^{1}H NMR spectrum of the product of hydrosilylation reaction of the 5th generation of poly(allyl)carbosilane dendrimer with 1,1,1,3,5,5,5-heptamethyltrisiloxane (G6(OTMS)).

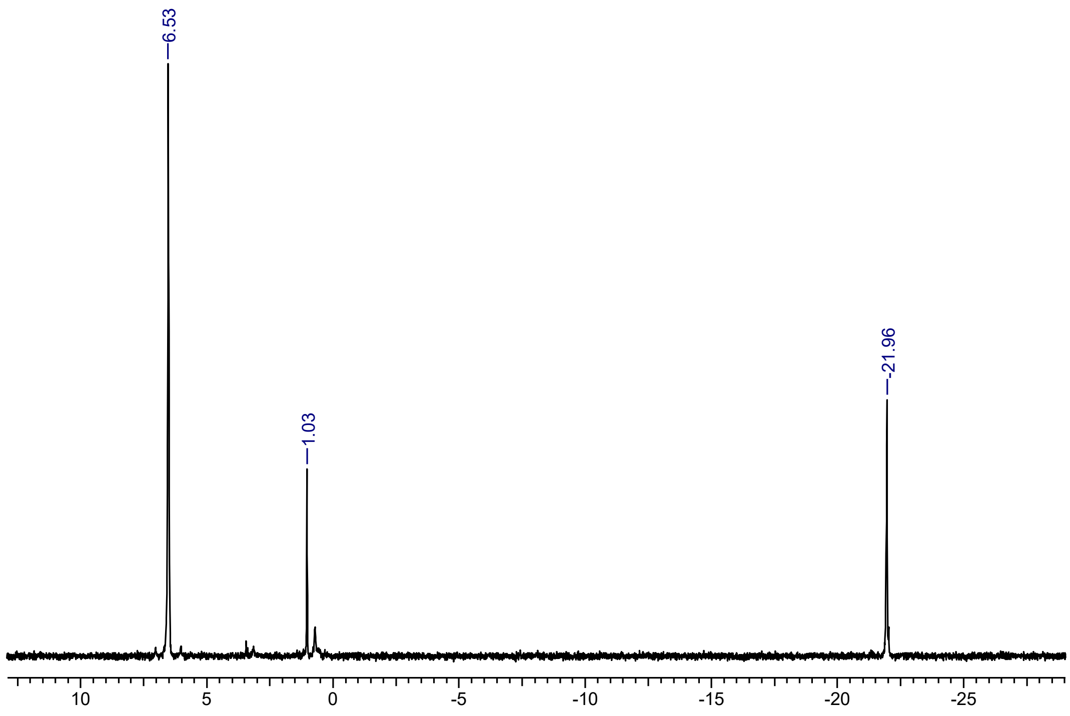

Figure S12. ^{29}Si NMR spectrum of the product of hydrosilylation reaction of the 5th generation of poly(allyl)carbosilane dendrimer with 1,1,1,3,5,5,5-heptamethyltrisiloxane (G6(OTMS)).

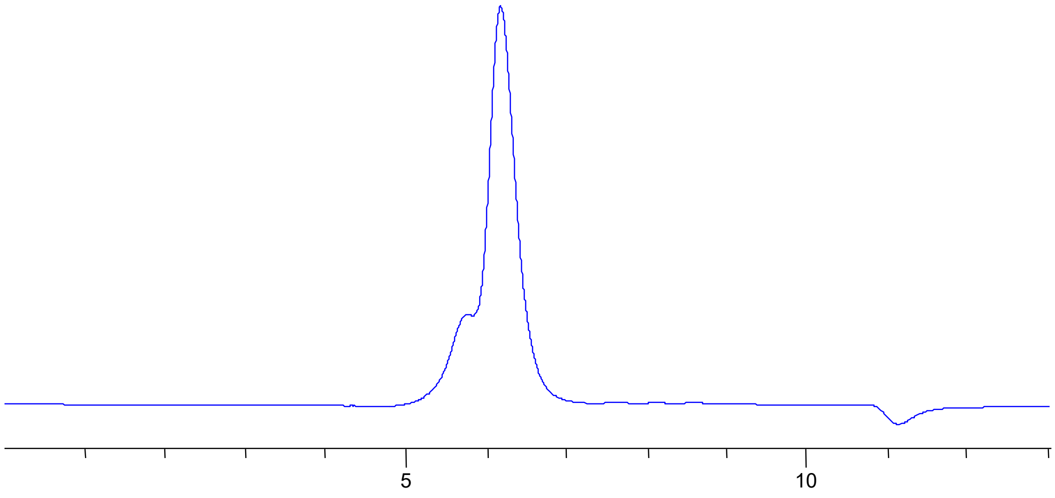

Figure S13. GPC curve of the 5th generation of poly(allyl)carbosilane dendrimer after the hydrosilylation reaction with 1,1,1,3,5,5,5-heptamethyltrisiloxane.

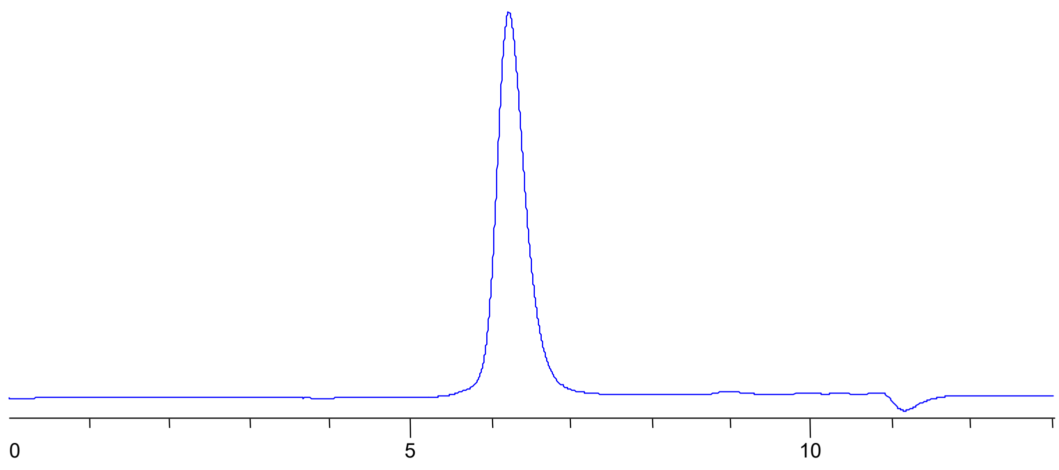

Figure S14. GPC curve of the 6th generation of carbosilane-siloxane dendrimer (G6(OTMS)) after preparative chromatography purification.

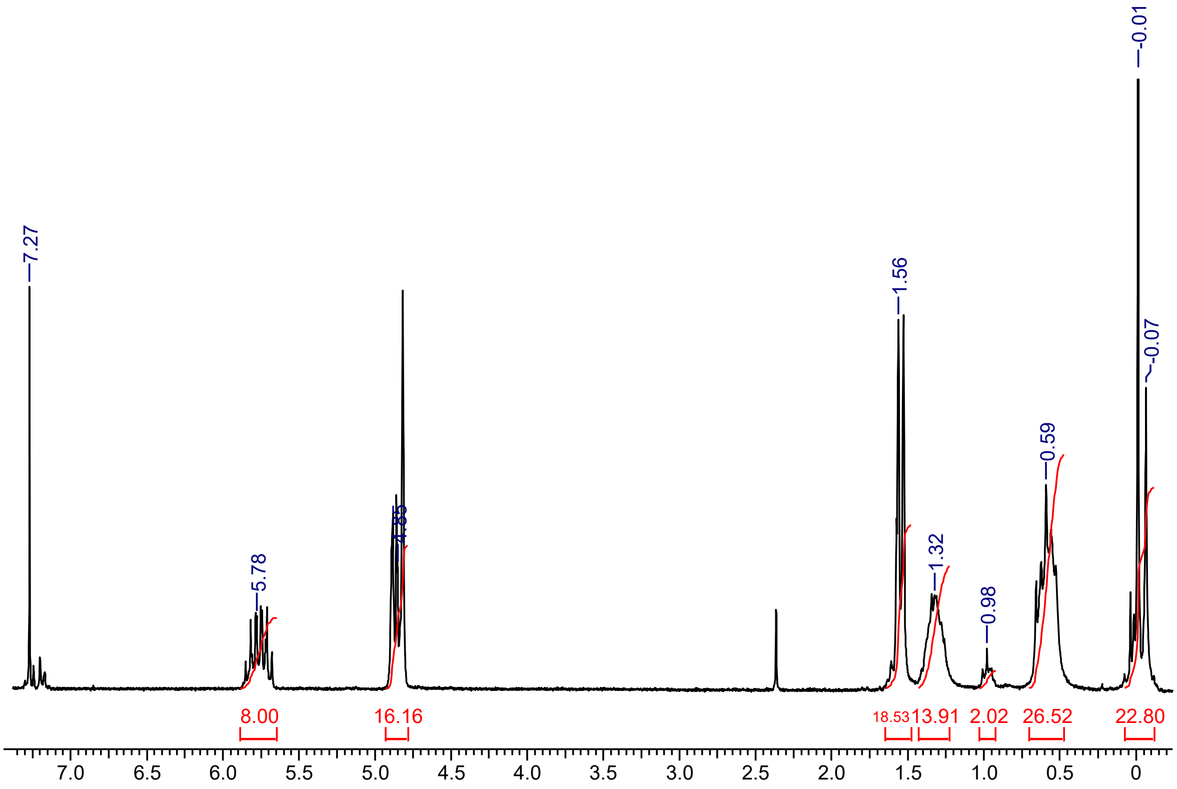

Figure S15. ^{1}H NMR spectrum of the 6th generation of poly(allyl)carbosilane dendrimer (G6(All)).

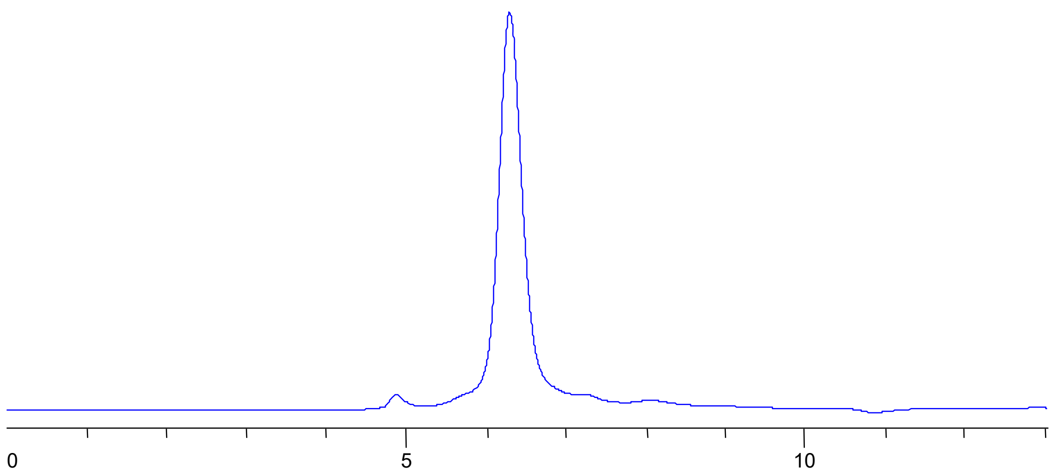

Figure S16. GPC curve of the 6th generation of poly(allyl)carbosilane dendrimer (G6(All)).

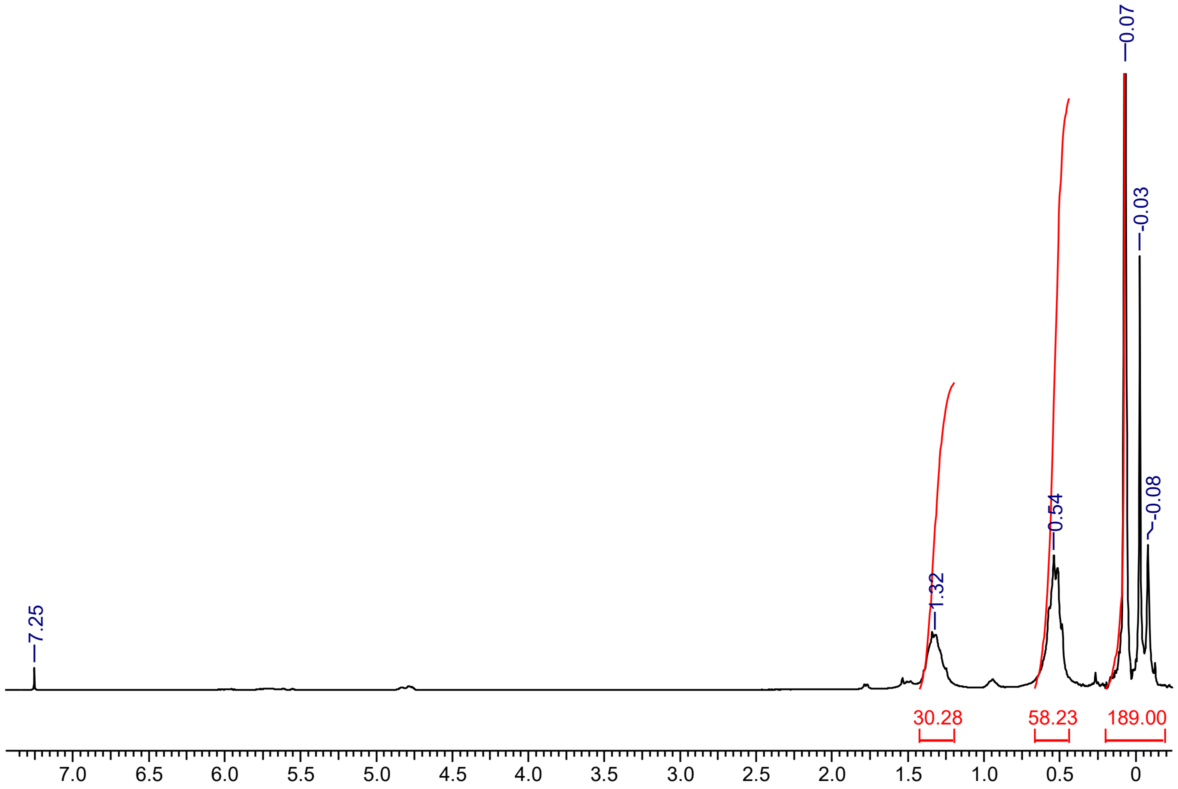

Figure S17. ^{1}H NMR spectrum of the product of hydrosilylation reaction of the 6th generation of poly(allyl)carbosilane dendrimer with 1,1,1,3,5,5,5-heptamethyltrisiloxane (G7(OTMS)).

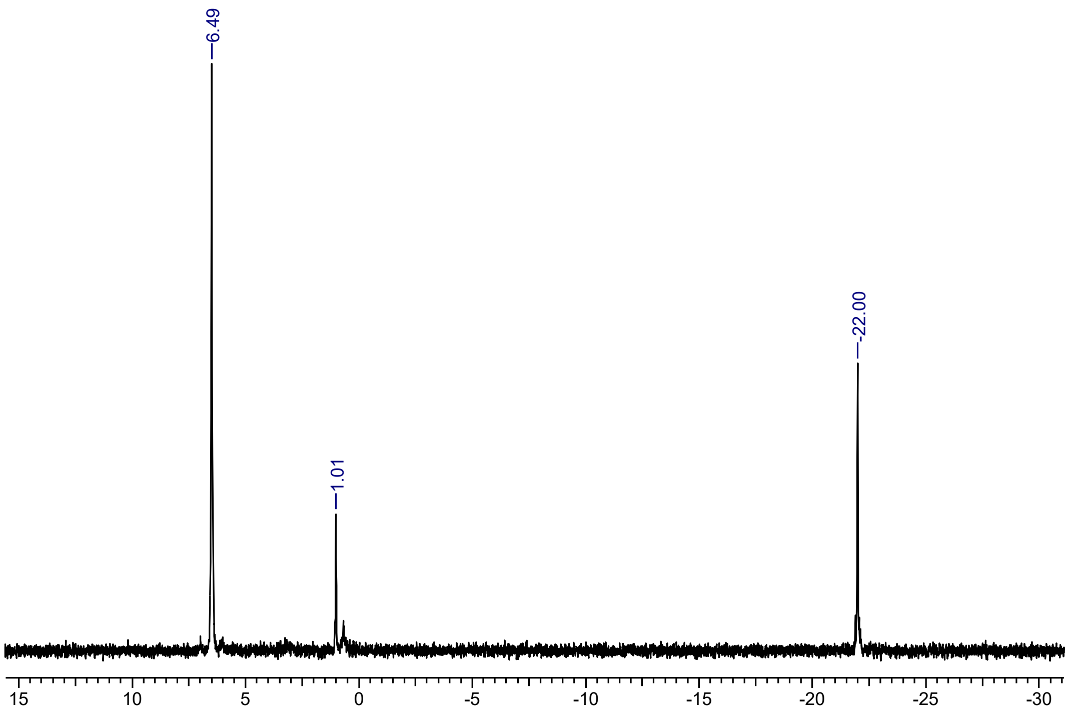

Figure S18. ^{29}Si NMR spectrum of the product of hydrosilylation reaction of the 6th generation of poly(allyl)carbosilane dendrimer with 1,1,1,3,5,5,5-heptamethyltrisiloxane (G7(OTMS)).

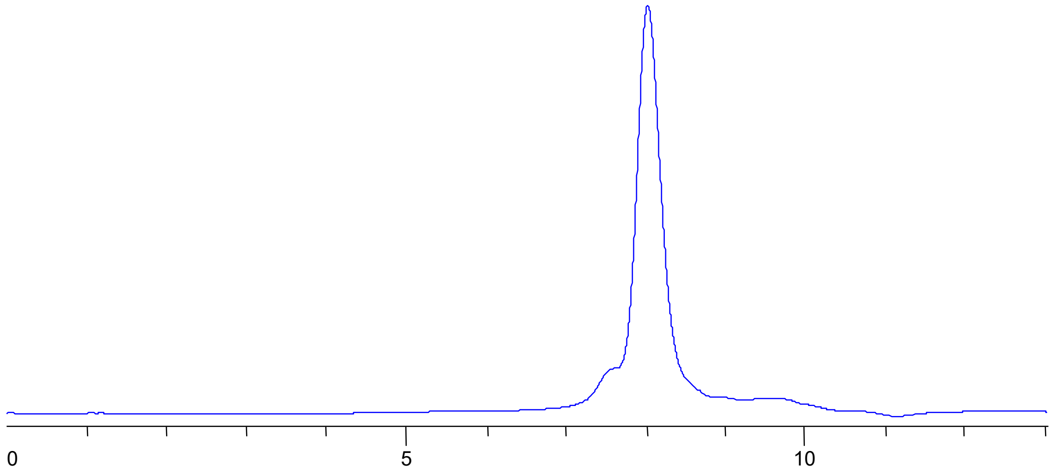

Figure S19. GPC curve of the 6th generation of poly(allyl)carbosilane dendrimer after the hydrosilylation reaction with 1,1,1,3,5,5,5-heptamethyltrisiloxane.

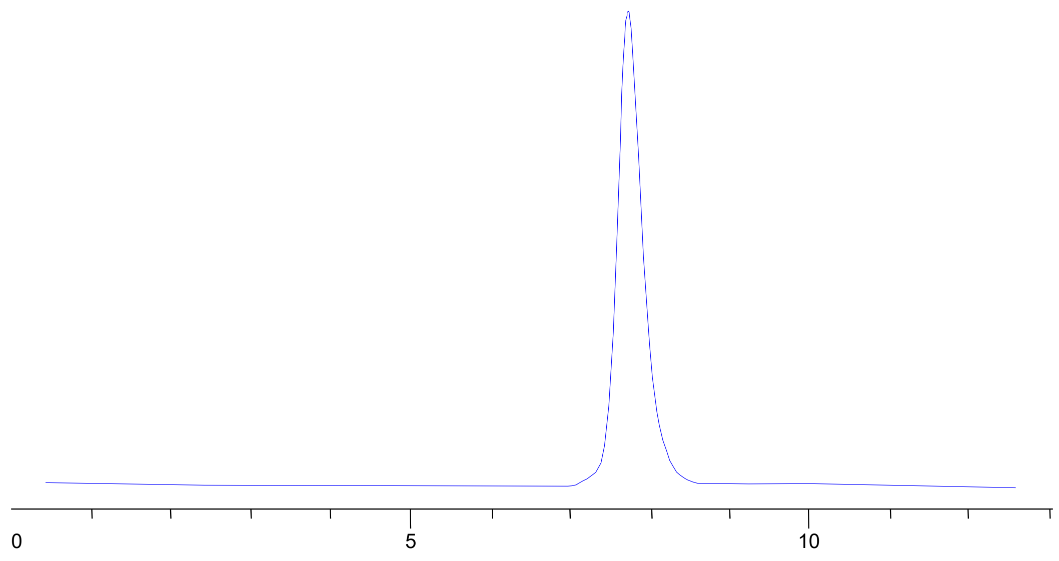

Figure S20. GPC curve of the 7th generation of carbosilane-siloxane dendrimer (G7(OTMS)) after preparative chromatography purification.

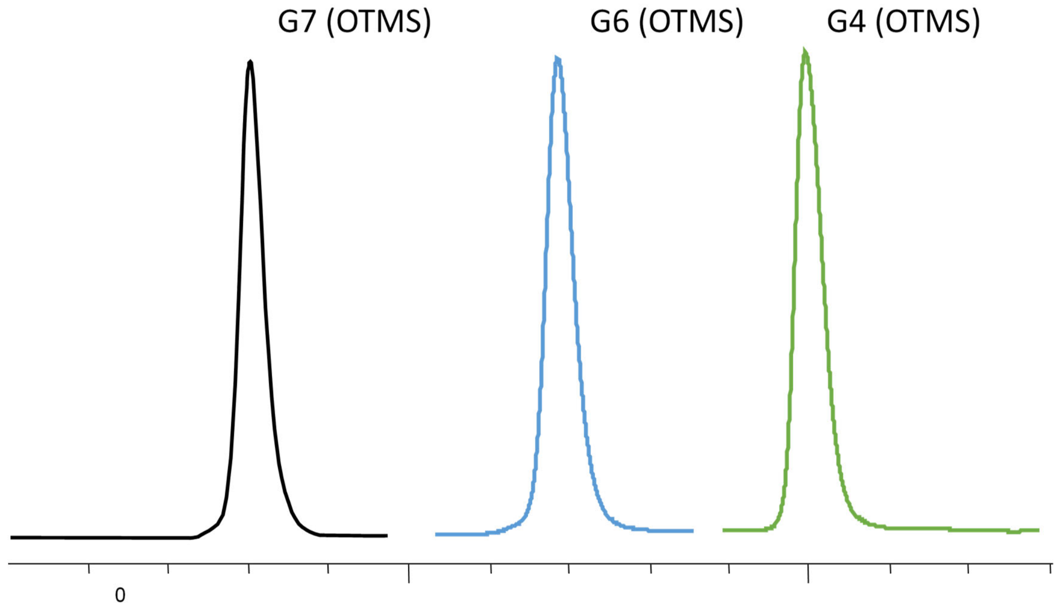

Figure 21. GPC curves of the 4th, 6th and 7th generations of carbosilane-siloxane dendrimers (G4(OTMS), G6(OTMS), G7(OTMS)) after preparative chromatography purification.

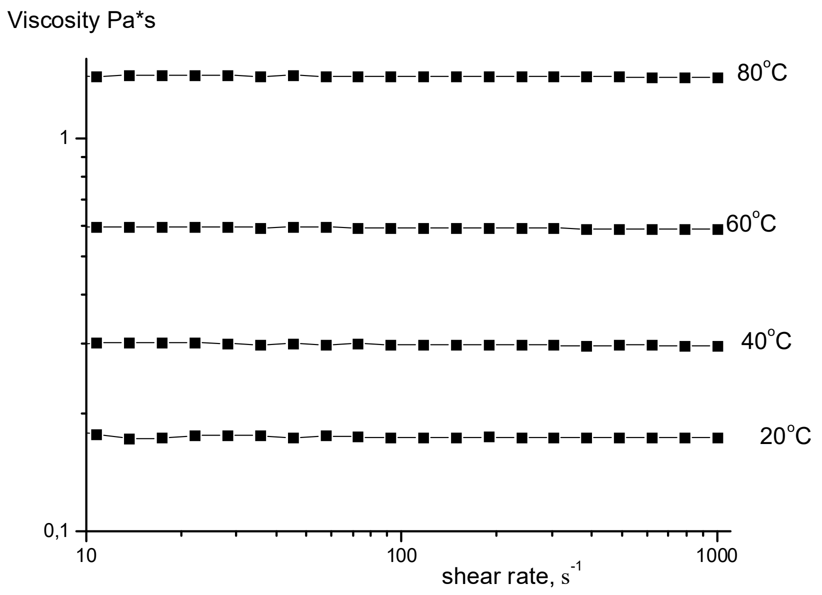

Figure S22. Flow curves for G4(OTMS) dendrimer melt at various temperatures.

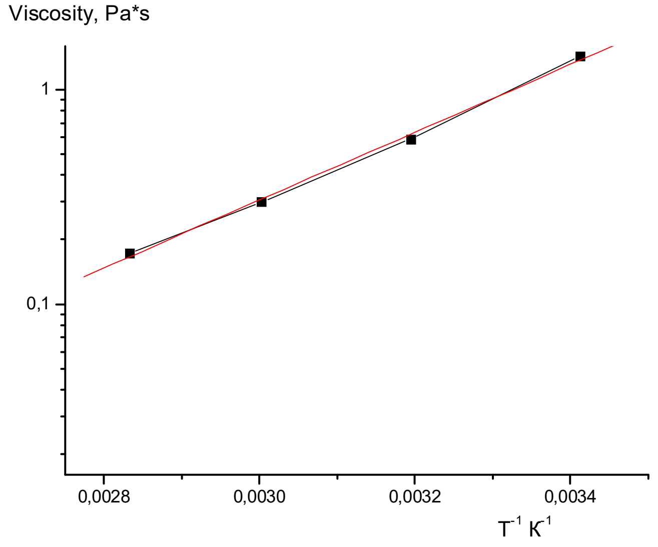

Figure S23. Temperature dependence of Newtonian viscosity of G4(OTMS) in Arrhenius coordinates.

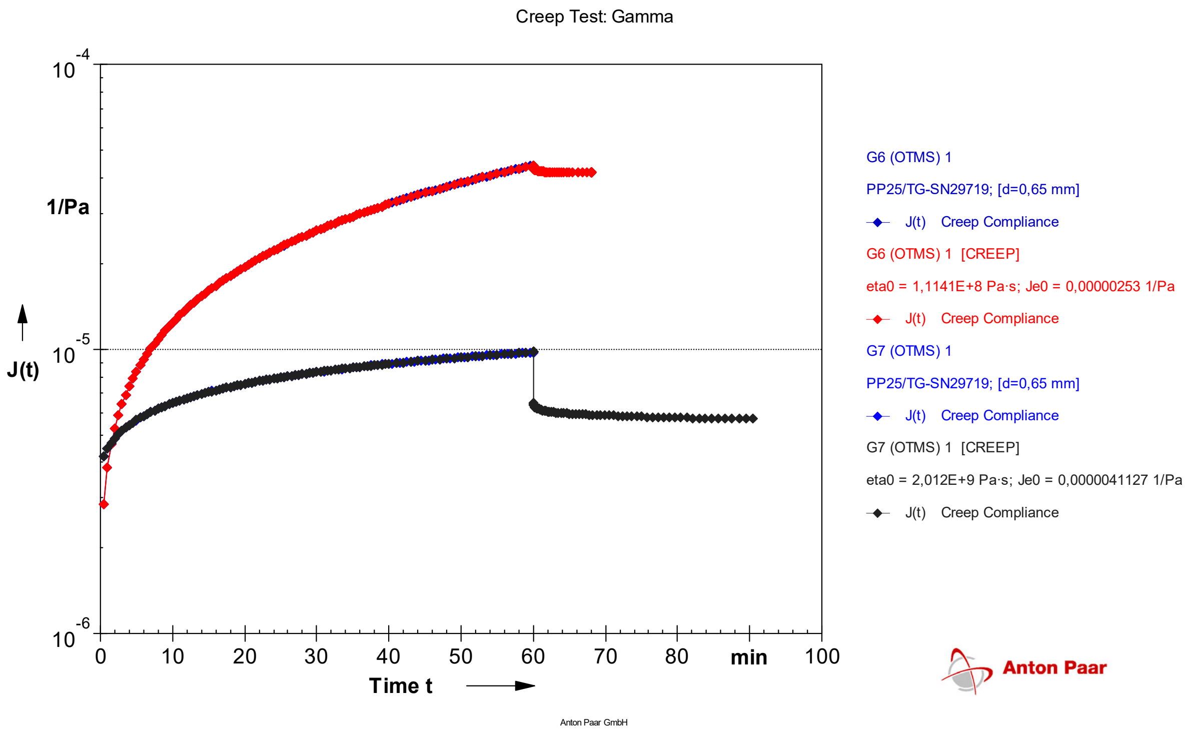

Figure S24. Creep compliance vs time for G6(OTMS) (dart blue curve) and G7(OTMS) (light blue curve). Shear stress τ = 10000 Pa. T = 25°C. Fitting with the Burgers equation is shown by red (G6(OTMS), fitting parameters: zero-shear viscosity η_0=1.11x10^8 Pa·s and the instantaneous creep compliance J0=2.5x10^{-6} 1/Pa) and black (G7(OTMS), fitting parameters: zero-shear viscosity η_0=2.01x10^9 Pa·s and the instantaneous creep compliance J0=4.1x10^{-6} 1/Pa).

Parameters of the force fields for the atoms used in MD simulations

Table S1. Bond potential $U_{bond} = \varepsilon_b(l - l_0)^2$.

Bond type	ε_b, kcal·mol^{-1}A^{-2}	l_0, A
Si-CH_3/Si-CH_2	238.0	1.809
O-Si	392.8	1.6650
CH_2-CH_2/CH_2-CH_3	322.761	1.526

Table S2. Valence angle potential $U_{angle} = \varepsilon_{angle}(\theta - \theta_0)^2$.

Atom type	ε_{angle}, kcal·mol^{-1}grad^{-2}	θ_0, grad
Si-O-Si	31.1	149.8
X-Si-X	44.4	113.5
X-CH_2-X	60.0	109.5

Table S3. Torsion angle potential $U_{tors} = \varepsilon_{tors}(1 + cos3\varphi)$.

Atom type	ε_{tors}, kcal·mol^{-1}
X-Si-CH_2-X	0.333
X-CH_2-CH_2-X	1.422

Table S4. Lennard-Jones potential: $U_{ij} = \varepsilon_{ij}\left[\left(\frac{R_{min,ij}}{r_{ij}}\right)^{12} - 2\left(\frac{R_{min,ij}}{r_{ij}}\right)^{6}\right]$.

$\varepsilon_{ij} = (\varepsilon_i \varepsilon_j)^{1/2}, \quad R_{min,ij} = 0.5(R_{min,i} + R_{min,j})$.

**Atom type**	**ε_i, kcal·mol^{-1}**	**R_{min}, A**
CH_2	0.1094	4.116
CH_3	0.1490	4.116
CH_3-**<u>Si</u>**-3CH_2	0.1900	4.450
CH_2,CH_3-**<u>Si</u>**-2O/3CH_3-**<u>Si</u>**-O	0.070	4.284
O	0.240	3.350

Table S5. Atomic masses and partial charges of the atoms.

**Atom type**	**m, am u**	**q, e**
CH_2,CH_3-**<u>Si</u>**-2O	28	0.640
3CH_3-**<u>Si</u>**-O	28	0.520
**<u>O</u>**	16	-0.440
**<u>CH3</u>**	15	-0.100
CH_3-**<u>Si</u>**-3CH_2	28	0.540
Si-**<u>CH_3</u>**	15	-0.135
CH_2-**<u>CH_2</u>**-CH_2 /CH_2-**<u>CH_2</u>**-CH_3	14	0.000

Coulomb potential: $U_q(r_{ij}) = \frac{q_i q_j}{r_{ij}} W_q(r_{ij})$, the screening function is as follows: $W_q(r) = \begin{cases} \left(1 - \frac{r}{R_q}\right)^2, & r < R_q \\ 0, & r \geq R_q \end{cases}$
